# Detecting the Candidate Gender Determinants by Bioinformatic Prediction of miRNAs and Their Targets from Transcriptome Sequences of the Male and Female Flowers in* Salix suchowensis*

**DOI:** 10.1155/2017/9614596

**Published:** 2017-05-30

**Authors:** Suyun Wei, Ning Ye, Tongming Yin

**Affiliations:** ^1^Co-Innovation Center for Sustainable Forestry in Southern China, Nanjing Forestry University, Nanjing, China; ^2^College of Information Science and Technology, Nanjing Forestry University, Nanjing, China

## Abstract

MicroRNAs (miRNAs) belong to a class of small, noncoding, and endogenous single-stranded RNAs that negatively regulate gene expression at the posttranscriptional level. Potential miRNAs can be identified based on sequence homology since miRNAs are highly conserved in plants. In this study, we aligned the expressed sequence tags derived from flower buds of male and female* S. suchowensis* to miRNAs in the miRBase, which enable us to identify 34 potential miRNAs from flower buds of the alternate sexes. Among them, 11 were from the female and 23 were from the male. Analyzing sequence complementarity led to identification of 124 and 55 miRNA targets in the male and female flower buds, respectively. By mapping the target genes of the predicted miRNAs to the sequence assemblies of* S. suchowensis*, a miR156 mediated gene was detected at the gender locus of willow, which was a transcription factor involved in flower development. It is noteworthy that this target is not expressed in male flower, while it is expressed fairly highly in female flower based on the transcriptome data derived from the alternate sexes of willows. This study provides new bioinformatic clue for further exploring the genetic mechanism underlying gender determination in willows.

## 1. Introduction

The miRNAs, approximately in length of 22-nucleotides, have been widely detected in diverse organisms. They are noncoding, endogenous, and single-stranded small RNAs, which negatively regulate gene expression in plants and animals at the posttranscriptional level [1, 2] by mediating target mRNA cleavage [3, 4] or translational repression [5, 6]. Increasing evidence suggests that plant miRNAs have several crucial roles in gene regulatory networks. They are believed to be involved in multiple plant developmental and metabolic pathways affecting developmental phase transitions [7–9], signal transduction [10], abiotic [11, 12] and biotic [13] stress responses, and phytohormone regulation [14]. The plant miRNAs were first discovered in* Arabidopsis thaliana* in 2002 [15–17]. Since then, considerable efforts have been exerted on identifying (e.g., [18–21]) and functionally annotating (e.g., [22–24]) the plant miRNAs. To date, 6,992 precursor miRNAs and 8,496 mature miRNAs from 73 plant species have been deposited in public miRNA database (miRBase 21.0, http://www.mirbase.org). Since miRNAs are highly conserved in plants, this database provides a useful platform for predicating miRNAs in other plants based on sequences homology.

The miRNAs have been found to be involved in regulating versatile biological processes, in particular, their role in regulating flower development has drawn much interest in the recent decades. For example, miR172 is found to act as a translational repressor of the* APETALA2* genes in the ABC model of flower development. The overexpression of miRNA172 results in early flowering and disrupts the floral organ differentiation in* A. thaliana* [25]. Besides miR172, miR164 in* A. thaliana* is found to target the* NAC* transcription factors, including* CUP*-*SHAPED COTYLEDON1 (CUC1)* and* CUC2*, which are redundantly required for the formation of boundaries that separate organ primordia; in particular, miR164c controls the number of flower petals by regulating the accumulation of* CUC1* and* CUC2* transcripts [26]. More recently, miR156 is found to target the* SPL* genes, which regulate floral transitions by modulating the expression of the key floral-promoting genes in* A. thaliana* [27] and maize [28]. Furthermore, miR319, miR159, miR164, and miR167 are found to have response for specifying particular cell types in the later stages of floral development [29–32].

Sex determination mechanism in plants has long been of considerable interest. Recent studies reveal that miRNAs also play an important role in determining the sexes of plants. The miRNAs regulating carpel and stamen development can ultimately lead to the production of unisexual or bisexual flowers. For instance, the* tasselseed4* miRNA (miR172) is found to be involved in the sex determination of the male inflorescence in maize [33, 34], which inhibits the translation of the* IDS1* transcript in the male florets. In the* ts4* or* ids1* mutants, female organs develop in the male inflorescence. Very recently, a study reported that a Y-specific sex-determinant candidate gene* (OGI)* was detected in persimmons* (Diospyros lotus)*.* OGI* encodes a small RNA that targets and represses the expression of the feminizing* male growth inhibitor (MeGI)* gene in male flowers [35]. The study reveals a mechanism that an autosomal feminizing gene suppressed by a Y-chromosome-encoded small RNA triggers the plant dioecy in which male is the heterogametic gender (XY).

Willows* (Salix)* are dioecious plants. The alternate sexes of willows bear morphologically different male or female catkins. A variety of studies presented reliable evidences that a single locus governed the sex determination in different willow species [36–40]. To date, all the mapping studies have confirmed that the female willows are the heterogametic gender, while the males are the homogametic sex, suggesting a ZW sex determination system in willows, and the gender locus is consistently mapped to the centromeric region on willow's chromosome XV [38–40]. With an international cooperative effort, the genome of* S. suchowensis* has been sequenced and the sequence scaffolds have been mapped along the 19 haploid chromosomes of willow [39, 41]. However, sequence assembly at the gender locus is fairly poor due to the high repetitive sequences in the centromeric region. In a previous study, we also analyzed the differentially expressed genes based on transcriptome sequencing of flower buds of the alternate sexes and detected 33 differentially expressed genes located on the incipient sex chromosome of Salicaceae [42]. Thus far, no obvious gender determinant has been identified based on the aforementioned studies. In consideration of the role of miRNAs in determining the sexes of plants, in this study, we predict the microRNAs and their targets from transcriptome sequences derived from male and female flowers of* S. suchowensis*. Our goal is to detect the candidate sex determinant of willow following the new perspective on role of miRNAs in sex determination of plants.

## 2. Methods

### 2.1. Sequence Resources

Reference miRNAs were downloaded from the publicly available miRBase database (http://www.mirbase.org/, Release 21, June 2014), which included 8,496 known mature miRNAs derived from 73 plant species. Duplicates in this database were manually culled out by an in-house multiple sequence alignment Perl script. The remaining unique mature miRNAs were used for homology search in the subsequent analysis. Transcriptome data were separately derived from flower buds of the alternate sexes of willow, which were deposit in the EMBL Nucleotide Archive (ENA, http://www.ebi.ac.uk/ena), with an ArrayExpress accession number of E-MTAB-1445. The transcriptome data totally contained 1,201,931 expressed sequence tags (ESTs) from both sexes, with an average length of 389 bp and a total length of 467.96 Mb. Among them, 629,854 and 572,089 ESTs were from the female and male flower buds, respectively.

### 2.2. Bioinformatic Prediction of Potential miRNAs

Prediction of miRNAs was performed based on sequence homology between the transcriptome sequences of willows and the screened miRNAs from the miRBase database following the method described by Zhang et al. [43]. Firstly, the unique mature miRNAs were aligned to the transcriptome sequences of the alternate sexes of willows by the Bowtie alignment toolkit [44]. In this analysis, the EST sequences with less than three nucleotide mismatches were chosen as candidate miRNA precursors. Subsequently, the candidate precursors were aligned to the NCBI nonredundant protein database (https://www.ncbi.nlm.nih.gov/) and RNA database Rfam (http://rfam.xfam.org/) to eliminate protein-coding sequences and noncoding RNAs (e.g., tRNA, rRNA, snRNA, or snoRNA) by BLAST search [45]. Finally, the remaining candidates from above analyses were further assessed by RNAfold from the ViennaRNA Package with default parameters [46], which produced parameters for examining the secondary hairpin structure, the Dicer cleavage site, and the minimum free energy (MFE) of the candidate sequences. Based on these parameters, potential miRNAs were manually identified according to the criteria for predicating plant miRNAs as described by Zhang et al. [43].

### 2.3. Analyzing the miRNA Targets

Bioinformatic prediction of the miRNA targets was conducted based on sequence complementarity by using the psRNATarget program with default parameters (http://plantgrn.noble.org/psRNATarget/) [47]. Annotation of the putative targets was performed by aligning the target sequences to NCBI nonredundant protein database (https://www.ncbi.nlm.nih.gov/) by BLAST searches. The annotated targets were assigned with confined GO terms in the UniProt database [48] and plotted by using the online analytical tool of WEGO (http://wego.genomics.org.cn/cgi-bin/wego/index.pl) [49]. The annotation information was used to retrieve keywords to identify genes related to sex determination and flowering.

The expression levels of miRNA targets in the male and female flowers were evaluated based on the number of reads captured from sequences of the targets. Sequence reads from flowers of the alternate sexes were separately aligned to the target sequences by using BWA (mismatch ≤ 2 bp, other parameters as default) [50]. And then, the SAM output from BWA was converted into a binary file (BAM) by using Samtools [51]. The number of mapped reads for each target gene was counted. Normalization of the mapped reads was estimated as transcripts per million (TPM) [52]. The heatmap for sex-specific expression profiling of each target was generated based on the log 2 TPM values by using R package [53].

Since the gender locus of willow was confirmedly mapped in the centromeric region of chromosome XV, we demonstrated the chromosomal location of each target by aligning them to the genome sequence assemblies of* S. suchowensis* with an in-house Perl script.

## 3. Results and Discussion

### 3.1. Bioinformatic Predication of miRNAs

A total of 8,496 mature plant miRNAs were downloaded from the miRBase database. After eliminating the duplicate miRNA sequences, we obtained 4,803 nonredundant reference miRNAs, which were used to search for homologs in the EST sequences derived from flower buds of alternate sexes. This analysis yielded 356 and 527 ESTs that were perfect or near-perfect matches for the reference miRNA sequences from the female and from the male, respectively. We then culled out the protein-coding sequences and noncoding RNAs such as tRNA, rRNA, snRNA, and snoRNA from the matched ESTs, and 117 ESTs from the female and 183 ESTs from the male remained for secondary structures analysis. Whether a stem-loop hairpin present in secondary structure of the sequences is a common feature of miRNA precursors, the ESTs failed to meet this criterion are eliminated. Finally, 11 miRNAs representing eight miRNAs families were identified in the female, including three miR162 members, two miR472 members, and one member for each of the remaining six miRNA families (Table 1), whereas, in the male flower buds, we identified 23 miRNAs from 11 miRNA families, including four miR6423 members, three miR167 members, and one or two members for each of the remaining families (Table 2).

**Figure 1:**
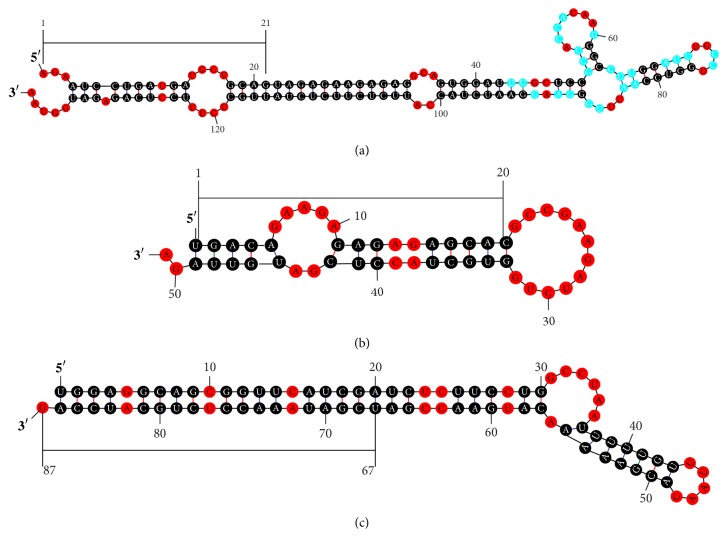
Three predicted stem-loop structures of pre-miRNAs identified in* S. suchowensis*. The mature miRNA is indicated with a black bar. (a) ssu-miR172a, whose precursor is in length of 138 bp. (b) ssu-miR156b, whose precursor is in length of 51 bp. (c) ssu-miR162b, whose precursor is in length of 87 bp.

Similar to animal miRNAs, plant miRNAs are normally in lengths of 20–24 bp and are processed from longer precursor transcripts [54]. The lengths of miRNAs identified in this study were in range of 20–22 bp, and the majority of them (18 miRNAs) were in length of 21 bp. In animals, the pre-miRNAs typically contain 70–80 nucleotides. By contrast, plant miRNA precursors are more variable in structure and size, ranging from 50 to 350 nucleotides or even longer. In some plants, the miRNA precursors were found to exceed 1000 nucleotides [54, 55]. In this study, the lengths of miRNA precursors were in range of 51–138 nucleotides, with an average of 85 nucleotides. All the mature ssu-miRNA sequences were located on the stem-arm of the stem-loop hairpin secondary structure of the potential pre-miRNAs. However, the location of mature miRNAs varied in different pre-miRNAs, such as ssu-miR156, ssu-miR160, ssu-miR166, ssu-miR172, and ssu-miR6423, the mature miRNA sequences located at the 5′ arm of the stem-loop hairpin. By contrast, the mature miRNA sequences in the remaining pre-miRNAs present at the 3′ arm of the stem-loop hairpin (Tables 1 and 2). All the pre-miRNAs identified in this study form stable stem-loop hairpins in their secondary structure (Figure 1).

### 3.2. Compare the miRNAs Identified in the Alternate Sexes

For the identified ssu-miRNAs (Table 3), they were classified into eight miRNA families in the female and classified into 11 miRNA families in the male. Five of the miRNA families were found to be common in the female and male flowers (i.e., ssu-miR162, ssu-miR169, ssu-miR472a, ssu-miR1448, and ssu-miR6243). By contrast, there were specific miRNA families (ssu-miR166, ssu-miR390) and miRNAs (ssu-miR172a) that were only detected in the female, and there were specific miRNA families (including ssu-miR156, ssu-miR160, ssu-miR167, ssu-miR396, ssu-miR7841, and ssu-miR396) and miRNAs (including ssu-miR162d, ssu-miR472c, and ssu-miR172b/c) that were only detected in the male.

In the identified ssu*-*miRNAs, five miRNA families (i.e., ssu-miR169, ssu-miR162, ssu-miR166, ssu-miR156, and ssu-miR396) were found to have the same sequences as that in more than 10 plant species. Complete matches to ssu-miR169, ssu-miR396, ssu-miR162, ssu-miR156, and ssu-miR166 sequences were detected in 58, 44, 35, 14, and 12 plant species, respectively (Table 3). The targets of these miRNA families were found to play important roles in flower development. For example, target of miR169 is a class-C gene that specify the identities of the reproductive organs during flower development in* Petunia hybrida* and* Antirrhinum majus* [56, 57], whereas miR156 is one of the most ancient miRNAs in perhaps all land plant lineages, which is a key regulator of vegetative phase change in* Arabidopsis*, maize, and rice [58]. Overexpression of miR156 results in prolonged expression of juvenile characters and extremely delayed flowering. Although most miRNAs are highly conserved in different plant species, specific miRNAs have been detected in Salicaceae species (Table 3). In this study, we also detected some Salicaceae-specific miRNAs (e.g., ssu-miR6423, ssu-miR472, ssu-miR1448, and ssu-miR7841) in* S. suchowensis*.

### 3.3. Analyzing the Targets of the Identified miRNAs

MiRNAs generally regulate the expression of specific genes via mRNA cleavage or translational inhibition. Based on the perfect or near-perfect sequence complementarity between miRNAs and their target transcripts, 55 targets (Table S1 in Supplementary Material available online at https://doi.org/10.1155/2017/9614596) for eight miRNAs from female flower buds and 124 targets (Table S2) for 13 miRNAs from male flower buds were detected separately (Figure S1). It showed that ssu-miR396 had the most targets (35), followed by ssu-miR472a (20) and ssu-miR156 (19).

Based on the GO terms of the corresponding homolog in the UniProt database [48], 44 (80%) and 96 (77%) targets from female (Table S3) and male (Table S4) flower buds were assigned with a confined GO term, respectively. The functionally annotated target genes belonged to categories of biological process, cellular component, and molecular function. Functional classification of the annotated targets was shown in a WEGO chart (Figure S2). In general, number of targets that have fallen in different component groups showed a similar pattern between the male and female. However, targets from the male were significantly enriched in some of the component groups, such as in component groups of envelope, antioxidant, enzyme regulator activity, molecular transducer, and cellular component biogenesis.

In order to gain more information for characterization of functional miRNAs involved in sex-specific flower development, we analyzed the expression profiles of the detected miRNA targets in male and female flower buds separately. Totally, 123 targets were found to be expressed in the male or female flower buds. Among them, only 8 targets are expressed in the female, only 8 targets are expressed in the male, and 107 targets are expressed in both male and female flower buds (Figure S3, Table S5). According to the TPM values, a negative correlation was observed between the expression of miRNAs and that of their targets. For some extremes, ssu-miR166, ssu-miR390, and ssu-miR172a were detected only in the female flower buds. Consequently, their targets had significantly lower expression level in female than in male flowers. In cases of ssu-miR156, ssu-miR162d, ssu-miR160, ssu-miR167, ssu-miR472c, ssu-miR172b/c, and ssu-miR7841, these miRNAs were only detected in the male, and their targets were found to be expressed significantly less in male than in female flowers. The negative correlation between the expression levels of miRNAs and those of their targets is in accordance with the gene silencing function of miRNAs.

### 3.4. Mapping the miRNA Targets along Chromosomal Assemblies of* S. suchowensis*

**Figure 2:**
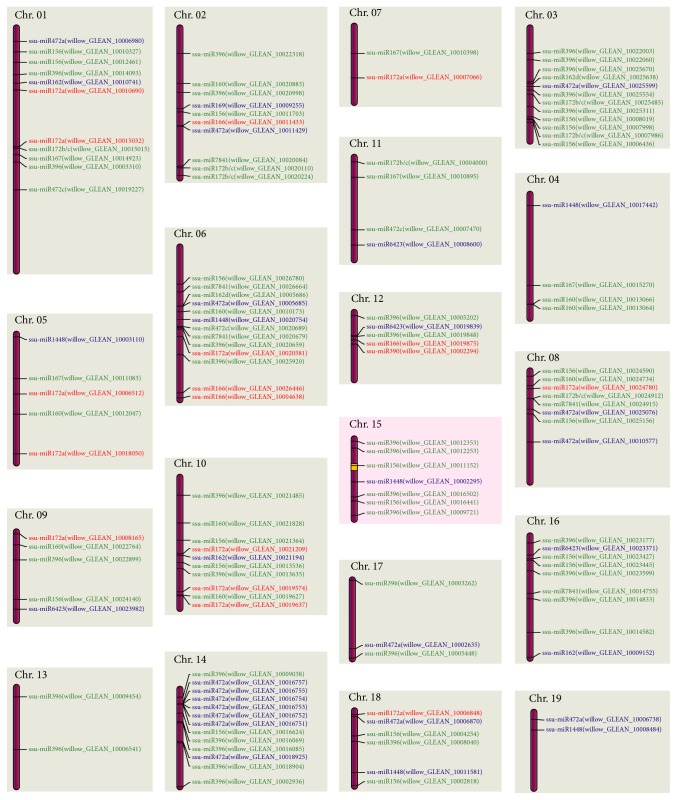
Distribution of miRNA targets on 19 chromosomes of* S. suchowensis*. Thirty target genes regulated by miRNAs commonly presented in both the female and the male flower buds are shown with blue, whereas 17 and 84 genes regulated by miRNAs presented exclusively in the female or the male are shown with red and green, respectively. In addition, the yellow block in chromosome XV represents gender locus in sex chromosome of* S. suchowensis*.

Based on the chromosomal assemblies established by Hou et al. [39], we plotted the distribution of the predicted targets along each chromosome in the willow genome. Totally, 47 targets detected in the female and 114 targets detected in the male were mapped onto the 19 haploid chromosomes in the genome of* S. suchowensis* (Figure 2, Table S6). Among them, 30 targets were regulated by miRNAs commonly presented in both the female and the male flower buds (in blue), 17 targets were regulated by female-specific miRNAs (in red), and 84 targets were regulated by male-specific miRNAs (in green). On many of the chromosomes, targets regulated by male-specific miRNAs were denser than those regulated by female-specific miRNAs.

The previous mapping study has confined the gender locus in a 742 Kb centromeric region on chromosome XV (the yellow block, Figure 2) [39]. It is notable that a target (willow_GLEAN_10011152) regulated by a male-specific miRNA (ssu-miR156) is located in the gender determination region. This target is expressed fairly highly in female flower buds, but no expression of it is observed in male flower buds (TPM: 576.4 versus 0). Furthermore, functional annotation of this target indicates that it is a transcription factor involved in the control of early flower development. Dedicated lab experiments need to be conducted to learn the biological role of these genes and their regulatory miRNA in willow sex determination. Salicaceae is a family of dioecious plants and possess the ZW sex determination system in which the female is the heterogametic gender [39, 59, 60]. Species with ZW sex determination system are relatively rare in high plants; thus willow provides a desirable system to learn the genetic mechanism underlying the ZW sex determination in plants. Although tremendous endeavor has been made to identify the sex-determining gene in Salicaceae species [38–40], no obvious candidate gene has been detected thus far. In this study, we explore the candidate in terms of miRNA perspective and detected an obvious candidate associated with flower development and sex differentiation of willow. However, this finding is discovered only based on the bioinformatic analyses; it need to be validated with experimental proof. Nevertheless, we identify a candidate sex determinant in willow by using bioinformatic analytical tools, and this finding is worthy of being explored by dedicated experimental efforts in future studies.

## 4. Conclusions

miRNAs are regulatory factors with important functions in diverse processes related to plant growth, development, morphogenesis, and stress responses. In particular, they are found to play important roles in regulating flower development, as well as sex differentiation of high plants. We analyzed the transcriptome sequences derived from the flower buds of the alternate sex of willow, focusing on predicating the putative miRNAs and their targets. Together with the genome sequence and mapping results of gender locus in willow, this work enables us to identify a candidate of sex determinant, which is a transcription factor involved in early flower development and the expression of it is only observed in female flower buds. Although we need to conduct a series of experimental studies to validate this bioinformatic finding, and the detailed mechanism on how the candidate regulates sex determination remains largely unknown; our work provides a valuable bioinformatic clue for further exploration to uncover the genetic mechanism underlying sex determination of willows, in which gender occurred through the ZW sex determination system.

## Supplementary Material

Figure S1: Number of targets for each miRNA in *S. suchowensis*. Green bars represent miRNAs common to female and male, whereas the red and blue bars correspond to miRNAs present exclusively in female or male, respectively.
Figure S2: Compare the number targets in different component groups between female and male flower buds of *S. suchowensis*.
Figure S3: Comparison of the expression levels of miRNA target genes between male and female buds of *S. suchowensis*. Color scales represent TPM normalized log2 transformed counts, whereas the red scales indicate high expression, and the blue scales indicate low expression.
Table S1: Predicted miRNA target genes in female flower buds of *S. suchowensis*.
Table S2: Predicted miRNA target genes in male flower buds of *S. suchowensis*.
Table S3: GO annotation of miRNA targets identified in female flower buds of *S. suchowensis*.
Table S4: GO annotation of miRNA targets identified in male flower buds of *S. suchowensis*.
Table S5: Different expression of miRNA targets between female and male flower buds of *S. suchowensis*.
Table S6: Distribution of miRNA target genes on 19 chromosomes of *S. suchowensis*.

## Figures and Tables

**Table 1:** Potential miRNAs identified in female flower buds of *S. suchowensis*.

miRNA	miRNA sequence (5′-3′)	LM (nt)	Arm	Pre-miR location	LP (nt)
ssu-miR162a	UCGAUAAACCUCUGCAUCCAG	21	3′	seq459792:217..303:+	86

ssu-miR162b	UCGAUAAACCUCUGCAUCCAG	21	3′	seq351845:128..215:−	87

ssu-miR162c	UCGAUAAACCUCUGCAUCCAG	21	3′	seq365695:26..113:+	87

ssu-miR166	GGAAUGUUGUCUGGCUCGAGG	21	5′	seq576601:133..255:+	122

ssu-miR169	CAGCCAAGGAUGACUUGCCGG	21	3′	seq292249:428..483:-	55
				seq292249:488..543:−	

ssu-miR390	CGCUAUCCAUCCUGAGUUUCA	21	3′	seq242088:345..447:−	102

ssu-miR172a	AGAAUCCUGAUGAUGCUGCAG	21	5′	seq288542:116..254:+	138
				seq263722:286..424:+	
				seq600570:208..346:−	

ssu-miR472a	UUUUCCCAACUCCACCCAUCCC	22	3′	seq62383:374..435:−	61

ssu-miR472b	UUUUCCCAACUCCACCCAUCCC	22	3′	seq355906:377..445:−	68

ssu-miR1448	CUUUCCAACGCCUCCCAUAC	20	3′	seq140125:105..174:+	69

ssu-miR6423	CCGCUGUCGCCACUAUCUUCCU	22	5′	seq431318:268..328:+	60
				seq593886:92..152:+	

LM: length of miRNA; Arm: location of mature miRNAs on secondary stem-loop structures of pre-miRNA sequences; Pre-miR location: miRNA precursor's location in ESTs of willow female flower buds; LP: length of pre-miRNA.

**Table 2:** Potential miRNAs identified in male flower buds of *S. suchowensis*.

miRNA	miRNA sequence (5′-3′)	LM (nt)	Arm	Pre-miR location	LP (nt)
ssu-miR156a	UGACAGAAGAUAGAGAGCAC	20	5′	seq517259:84..165:+	81
ssu-miR156b	UGACAGAAGAUAGAGAGCAC	20	5′	seq325710:29..80:−	51
ssu-miR160	UGCCUGGCUCCCUGUAUGCC	20	5′	seq145824:369..444:−	75
ssu-miR167a	AGAUCAUGUGGCAGUUUCACC	21	3′	seq137705:68..140:+	72
				seq383115:96..168:+	
ssu-miR167b	AGAUCAUGUGGCAGUUUCACC	21	3′	seq450474:97..169:+	72
ssu-miR167c	AGAUCAUGUGGCAGUUUCACC	21	3′	seq460555:421..495:−	74
				seq460555:481..555:−	
ssu-miR162a	UCGAUAAACCUCUGCAUCCAG	21	3′	seq430376:217..303:+	86
ssu-miR162d	UGGAGGCAGCGGUUCAUCGAUC	22	5′	seq344645:410..500:+	90
ssu-miR169a	CAGCCAAGGAUGACUUGCCGG	21	5′	seq159785:89..210:−	121
ssu-miR169b	CAGCCAAGGAUGACUUGCCGG	21	5′	seq167083:107..214:+	107
				seq166207:31..138:+	
ssu-miR172b	GCAGCACCAUCAAGAUUCACA	21	5′	seq555414:28..136:+	108
ssu-miR172c	GCAGCACCAUCAAGAUUCACA	21	5′	seq562308:14..122:−	108
ssu-miR396a	UUCCACAGCUUUCUUGAACUG	21	3′	seq114772:89..196:+	107
ssu-miR396b	UUCCACAGCUUUCUUGAACUG	21	3′	seq307347:218..305:−	87
ssu-miR472a	UUUUCCCAACUCCACCCAUCCC	22	3′	seq467610:63..125:+	62
ssu-miR472c	UCUUGCCUACUCCUCCCAUUCC	22	3′	seq106361:38..109:+	71
ssu-miR1448a	CUUUCCAACGCCUCCCAUAC	20	3′	seq106361:219..287:+	68
ssu-miR1448b	CUUUCCAACGCCUCCCAUAC	20	3′	seq83628:392..461:−	69
				seq83628:422..491:−	
ssu-miR7841	GGGGGUUGCUGUCAAGCAUAA	21	3′	seq306990:54..154:−	100
ssu-miR6423a	CCGCUGUCGCCACUAUCUUCCU	22	5′	seq268392:35..125:+	90
ssu-miR6423b	CCGCUGUCGCCACUAUCUUCCU	22	5′	seq228745:13..103:+	90
ssu-miR6423c	CCGCUGUCGCCACUAUCUUCCU	22	5′	seq199601:0..96:+	96
ssu-miR6423d	CCGCUGUCGCCACUAUCUUCCU	22	5′	seq100248:45..105:−	60
				seq148917:45..105:−	

LM: length of miRNA; Arm: location of mature miRNAs on secondary stem-loop structures of pre-miRNA sequences; Pre-miR location: miRNA precursor's location in ESTs of willow male flower buds; LP: length of pre-miRNA.

**Table 3:** Summary of miRNAs identified in flower budsof the alternate sexes in *S. suchowensis*.

Name	miRNA family	miRNA sequence (5′-3′)	Number of species
Common in female and male	ssu-miR6423	CCGCUGUCGCCACUAUCUUCCU	1
	ssu-miR169	CAGCCAAGGAUGACUUGCCGG	58
	ssu-miR162	UCGAUAAACCUCUGCAUCCAG	35
	ssu-miR472a	UUUUCCCAACUCCACCCAUCCC	1
	ssu-miR1448	CUUUCCAACGCCUCCCAUAC	1

Female-specific	ssu-miR166	GGAAUGUUGUCUGGCUCGAGG	12
	ssu-miR390	CGCUAUCCAUCCUGAGUUUCA	5
	ssu-miR172a	AGAAUCCUGAUGAUGCUGCAG	3

Male-specific	ssu-miR156	UGACAGAAGAUAGAGAGCAC	14
	ssu-miR162d	UGGAGGCAGCGGUUCAUCGAUC	3
	ssu-miR160	UGCCUGGCUCCCUGUAUGCC	6
	ssu-miR7841	GGGGGUUGCUGUCAAGCAUAA	1
	ssu-miR167	AGAUCAUGUGGCAGUUUCACC	4
	ssu-miR396	UUCCACAGCUUUCUUGAACUG	44
	ssu-miR472c	UCUUGCCUACUCCUCCCAUUCC	1
	ssu-miR172b/c	GCAGCACCAUCAAGAUUCACA	2

Number of species indicates in how many plant species the homologous miRNA was detected.
