# Supplementary material for: Detecting the Candidate Gender Determinants by Bioinformatic Prediction of miRNAs and Their Targets from Transcriptome Sequences of the Male and Female Flowers in Salix suchowensis
Source: Biomed Res Int. 2017 May 30;2017:9614596. doi: 10.1155/2017/9614596 (PMC5468582; doi:10.1155/2017/9614596)
Supplement: Supplementary file 1 — Figure S1: Number of targets for each miRNA in S. suchowensis. Green bars represent miRNAs common to female and male, whereas the red and blue bars correspond to miRNAs present exclusively in female or male, respectively. Figure S2: Compare the number targets in different component groups between female and male flower buds of S. suchowensis. Figure S3: Comparison of the expression levels of miRNA target genes between male and female buds of S. suchowensis. Color scales represent TPM normalized log2 transformed counts, whereas the red scales indicate high expression, and the blue scales indicate low expression. Table S1: Predicted miRNA target genes in female flower buds of S. suchowensis. Table S2: Predicted miRNA target genes in male flower buds of S. suchowensis. Table S3: GO annotation of miRNA targets identified in female flower buds of S. suchowensis. Table S4: GO annotation of miRNA targets identified in male flower buds of S. suchowensis. Table S5: Different expression of miRNA targets between female and male flower buds of S. suchowensis. Table S6: Distribution of miRNA target genes on 19 chromosomes of S. suchowensis. [file 9614596.f1.zip › Supplementary Materials/Table S2.doc]

**Table S2.** Predicted miRNA target genes in male flower buds of *S. suchowensis*.

| **miRNA_Acc.** | **Target_Acc.** | **Expectation** | **miRNAstart** | **miRNAend** | **Targetstart** | **Target**  **end** | **miRNA_aligned_fragment** | **Target_aligned_fragment** | **Inhibition** | **Target_Description** |
| --- | --- | --- | --- | --- | --- | --- | --- | --- | --- | --- |
| ssu-miR1448 | willow_GLEAN_10002295 | 2 | 1 | 20 | 17 | 37 | CUUUCCAACGCCUCCCAU-AC | GUCGUGGGAGGUGUUGGAAAG | Cleavage | [mRNA] locus=scaffold349:7165:10818:- |
| ssu-miR1448 | willow_GLEAN_10017442 | 2.5 | 1 | 20 | 4093 | 4112 | CUUUCCAACGCCUCCCAUAC | CUAUGGGAGGCUUUGGAGAG | Translation | [mRNA] locus=scaffold25:1904834:1939268:+ |
| ssu-miR1448 | willow_GLEAN_10017442 | 2.5 | 1 | 20 | 8029 | 8048 | CUUUCCAACGCCUCCCAUAC | CUAUGGGAGGCUUUGGAGAG | Translation | [mRNA] locus=scaffold25:1904834:1939268:+ |
| ssu-miR1448 | willow_GLEAN_10008484 | 2.5 | 1 | 20 | 581 | 600 | CUUUCCAACGCCUCCCAUAC | GUAUGGCAGGUCUUGGAAAG | Translation | [mRNA] locus=scaffold95:686862:688225:+ |
| ssu-miR1448 | willow_GLEAN_10001511 | 2.5 | 1 | 20 | 692 | 711 | CUUUCCAACGCCUCCCAUAC | GUAUGGGUGGAGUUGGGAAG | Translation | [mRNA] locus=scaffold01959:528:3584:+ |
| ssu-miR1448 | willow_GLEAN_10011581 | 3 | 1 | 20 | 1604 | 1623 | CUUUCCAACGCCUCCCAUAC | GUAUGGGAGGAGUUGGGAUG | Translation | [mRNA] locus=scaffold58:258675:263128:- |
| ssu-miR1448 | willow_GLEAN_10020754 | 3 | 1 | 20 | 1443 | 1463 | CUUUCCAAC-GCCUCCCAUAC | UUAUGGGAUGCAGUUGGAAAG | Translation | [mRNA] locus=scaffold13:2484340:2486208:+ |
| ssu-miR1448 | willow_GLEAN_10003110 | 3 | 1 | 20 | 497 | 516 | CUUUCCAACGCCUCCCAUAC | GUAUGGAAGGACUUGGAAAG | Translation | [mRNA] locus=scaffold234:115330:116082:- |
| ssu-miR156 | willow_GLEAN_10023445 | 1 | 1 | 20 | 857 | 876 | UGACAGAAGAUAGAGAGCAC | GUGCUCUCUCUCUUCUGUCA | Translation | [mRNA] locus=scaffold6:2263338:2265455:- |
| ssu-miR156 | willow_GLEAN_10010327 | 1 | 1 | 20 | 806 | 825 | UGACAGAAGAUAGAGAGCAC | GUGCUCUCUCUCUUCUGUCA | Translation | [mRNA] locus=scaffold72:480267:483403:- |
| ssu-miR156 | willow_GLEAN_10016441 | 1 | 1 | 20 | 902 | 921 | UGACAGAAGAUAGAGAGCAC | GUGCUCUCUCUCUUCUGUCA | Translation | [mRNA] locus=scaffold29:1155624:1158040:+ |
| ssu-miR156 | willow_GLEAN_10016624 | 1 | 1 | 20 | 824 | 843 | UGACAGAAGAUAGAGAGCAC | GUGCUCUCUCUCUUCUGUCA | Translation | [mRNA] locus=scaffold28:654659:657322:+ |
| ssu-miR156 | willow_GLEAN_10002818 | 1 | 1 | 20 | 1073 | 1092 | UGACAGAAGAUAGAGAGCAC | GUGCUCUCUCUCUUCUGUCA | Translation | [mRNA] locus=scaffold257:65931:68026:- |
| ssu-miR156 | willow_GLEAN_10023427 | 1 | 1 | 20 | 1217 | 1236 | UGACAGAAGAUAGAGAGCAC | GUGCUCUCUCUCUUCUGUCA | Translation | [mRNA] locus=scaffold6:2104775:2108992:+ |
| ssu-miR156 | willow_GLEAN_10007998 | 1 | 1 | 20 | 1154 | 1173 | UGACAGAAGAUAGAGAGCAC | GUGCUCUCUCUCUUCUGUCA | Translation | [mRNA] locus=scaffold103:354717:358342:+ |
| ssu-miR156 | willow_GLEAN_10013536 | 1 | 1 | 20 | 1049 | 1068 | UGACAGAAGAUAGAGAGCAC | GUGCUCUCUCUCUUCUGUCA | Translation | [mRNA] locus=scaffold44:214265:216959:+ |
| ssu-miR156 | willow_GLEAN_10025156 | 1 | 1 | 20 | 1091 | 1110 | UGACAGAAGAUAGAGAGCAC | GUGCUCUCUCUCUUCUGUCA | Translation | [mRNA] locus=scaffold4:4653038:4655786:- |
| ssu-miR156 | willow_GLEAN_10026780 | 1 | 1 | 20 | 1286 | 1305 | UGACAGAAGAUAGAGAGCAC | GUGCUCUCUCUCUUCUGUCA | Translation | [mRNA] locus=scaffold1:2103103:2108967:+ |
| ssu-miR156 | willow_GLEAN_10008019 | 1 | 1 | 20 | 2993 | 3012 | UGACAGAAGAUAGAGAGCAC | GUGCUCUCUCUCUUCUGUCA | Translation | [mRNA] locus=scaffold103:566844:587312:- |
| ssu-miR156 | willow_GLEAN_10011152 | 2 | 1 | 20 | 887 | 906 | UGACAGAAGAUAGAGAGCAC | GUGCUCACUCUCUUCUGUCA | Translation | [mRNA] locus=scaffold64:726048:729308:+ |
| ssu-miR156 | willow_GLEAN_10012461 | 2.5 | 1 | 20 | 397 | 416 | UGACAGAAGAUAGAGAGCAC | AUGCUUUCUGUCUUCUGUUA | Cleavage | [mRNA] locus=scaffold52:601329:604706:- |
| ssu-miR156 | willow_GLEAN_10011703 | 2.5 | 1 | 20 | 983 | 1002 | UGACAGAAGAUAGAGAGCAC | AUGCUUUCUCUCUUCUGUCA | Translation | [mRNA] locus=scaffold57:173690:178509:- |
| ssu-miR156 | willow_GLEAN_10004254 | 2.5 | 1 | 20 | 467 | 486 | UGACAGAAGAUAGAGAGCAC | GUGCUCUUUUUCUUCUGUCC | Translation | [mRNA] locus=scaffold186:121141:126875:- |
| ssu-miR156 | willow_GLEAN_10021364 | 3 | 1 | 20 | 935 | 955 | UGACAGAAGAUAGA-GAGCAC | AUGCUCCUCUAUCUUUUGUUA | Cleavage | [mRNA] locus=scaffold11:2181352:2182701:- |
| ssu-miR156 | willow_GLEAN_10024590 | 3 | 1 | 20 | 298 | 317 | UGACAGAAGAUAGAGAGCAC | CUGCUCUCUGCCUUCUGUUA | Translation | [mRNA] locus=scaffold4:576593:579568:+ |
| ssu-miR156 | willow_GLEAN_10006436 | 3 | 1 | 20 | 1434 | 1453 | UGACAGAAGAUAGAGAGCAC | GAGGUUUCUAUCUUCUGUUA | Cleavage | [mRNA] locus=scaffold130:27909:34462:+ |
| ssu-miR156 | willow_GLEAN_10024140 | 3 | 1 | 20 | 881 | 900 | UGACAGAAGAUAGAGAGCAC | GUGAUGUCUAUCUUUUGUUA | Cleavage | [mRNA] locus=scaffold5:2279830:2281875:+ |
| ssu-miR160 | willow_GLEAN_10024734 | 0 | 1 | 20 | 1405 | 1424 | UGCCUGGCUCCCUGUAUGCC | GGCAUACAGGGAGCCAGGCA | Cleavage | [mRNA] locus=scaffold4:1597517:1600625:+ |
| ssu-miR160 | willow_GLEAN_10013064 | 0 | 1 | 20 | 1306 | 1325 | UGCCUGGCUCCCUGUAUGCC | GGCAUACAGGGAGCCAGGCA | Cleavage | [mRNA] locus=scaffold47:555671:559308:+ |
| ssu-miR160 | willow_GLEAN_10022764 | 0 | 1 | 20 | 1318 | 1337 | UGCCUGGCUCCCUGUAUGCC | GGCAUACAGGGAGCCAGGCA | Cleavage | [mRNA] locus=scaffold7:707175:710666:+ |
| ssu-miR160 | willow_GLEAN_10019627 | 0 | 1 | 20 | 1237 | 1256 | UGCCUGGCUCCCUGUAUGCC | GGCAUACAGGGAGCCAGGCA | Cleavage | [mRNA] locus=scaffold16:2368416:2370904:- |
| ssu-miR160 | willow_GLEAN_10010173 | 0.5 | 1 | 20 | 1372 | 1391 | UGCCUGGCUCCCUGUAUGCC | GGCAUGCAGGGAGCCAGGCA | Cleavage | [mRNA] locus=scaffold74:451710:454375:+ |
| ssu-miR160 | willow_GLEAN_10020883 | 0.5 | 1 | 20 | 1309 | 1328 | UGCCUGGCUCCCUGUAUGCC | GGCAUGCAGGGAGCCAGGCA | Cleavage | [mRNA] locus=scaffold12:870221:873000:+ |
| ssu-miR160 | willow_GLEAN_10012047 | 0.5 | 1 | 20 | 1306 | 1325 | UGCCUGGCUCCCUGUAUGCC | GGCAUGCAGGGAGCCAGGCA | Cleavage | [mRNA] locus=scaffold54:526535:529408:+ |
| ssu-miR160 | willow_GLEAN_10013066 | 3 | 1 | 20 | 91 | 112 | UGCCUGGC-UCCC-UGUAUGCC | GACAUACAAGGGAUGCCAGGCA | Cleavage | [mRNA] locus=scaffold47:579335:581490:+ |
| ssu-miR160 | willow_GLEAN_10021828 | 3 | 1 | 20 | 2732 | 2751 | UGCCUGGCUCCCUGUAUGCC | GGCAGAUGGAGAGCCAGGCA | Translation | [mRNA] locus=scaffold10:3414912:3421552:- |
| ssu-miR162 | willow_GLEAN_10010741 | 3 | 1 | 21 | 1266 | 1286 | UCGAUAAACCUCUGCAUCCAG | CUGGAUGCAGAGCUUUAUUAA | Translation | [mRNA] locus=scaffold67:765160:772113:+ |
| ssu-miR162 | willow_GLEAN_10001417 | 3 | 1 | 21 | 643 | 664 | UCGAUAAACCUC-UGCAUCCAG | UUGGAUUCAAGAGGUUUAUUGG | Cleavage | [mRNA] locus=scaffold02283:1967:3118:- |
| ssu-miR162 | willow_GLEAN_10009152 | 3 | 1 | 21 | 402 | 422 | UCGAUAAACCUCUGCAUCCAG | UUCGGUGAAGAGGUUUAUCGG | Cleavage | [mRNA] locus=scaffold86:85794:89433:+ |
| ssu-miR162 | willow_GLEAN_10021194 | 3 | 1 | 21 | 1912 | 1932 | UCGAUAAACCUCUGCAUCCAG | CUGGAUCCAGAGUAUUAUCGA | Translation | [mRNA] locus=scaffold11:552293:554819:- |
| ssu-miR162d | willow_GLEAN_10025638 | 2.5 | 1 | 22 | 1284 | 1305 | UGGAGGCAGCGGUUCAUCGAUC | UAUUGAUGGAGUGCUGCCUCCA | Cleavage | [mRNA] locus=scaffold3:3504571:3508364:+ |
| ssu-miR162d | willow_GLEAN_10005686 | 3 | 1 | 22 | 3108 | 3130 | UGGAGGCAGC-GGUUCAUCGAUC | GAUGGGUGAACUUGCUGCCUCCA | Translation | [mRNA] locus=scaffold144:149955:156232:+ |
| ssu-miR167 | willow_GLEAN_10014923 | 2 | 1 | 21 | 372 | 392 | AGAUCAUGUGGCAGUUUCACC | AGUGAAACUGCCAUAUGACCU | Cleavage | [mRNA] locus=scaffold36:172529:177664:- |
| ssu-miR167 | willow_GLEAN_10011083 | 2.5 | 1 | 21 | 217 | 238 | AGAUCAUGUGGCA-GUUUCACC | GGUGAAACCUGGCACAUGGUCU | Translation | [mRNA] locus=scaffold65:922006:924358:- |
| ssu-miR167 | willow_GLEAN_10010398 | 2.5 | 1 | 21 | 310 | 331 | AGAUCAUGUGGC-AGUUUCACC | GGUGAAACUUGGCACAUGGUCU | Translation | [mRNA] locus=scaffold73:339213:341398:+ |
| ssu-miR167 | willow_GLEAN_10010895 | 3 | 1 | 21 | 1582 | 1602 | AGAUCAUGUGGCAGUUUCACC | UCUGAAACUGCCACACGAUUU | Cleavage | [mRNA] locus=scaffold66:464500:472509:+ |
| ssu-miR167 | willow_GLEAN_10015270 | 3 | 1 | 21 | 3940 | 3961 | AGAUCAUGU-GGCAGUUUCACC | UGUGAAGCUGCAGAUAUGAUCU | Translation | [mRNA] locus=scaffold34:472555:481178:+ |
| ssu-miR169 | willow_GLEAN_10009255 | 3 | 1 | 21 | 2660 | 2680 | CAGCCAAGGAUGACUUGCCGG | UUGGAAAGUCAUCCUUGGUUC | Cleavage | [mRNA] locus=scaffold85:164613:181596:+ |
| ssu-miR172 | willow_GLEAN_10015015 | 2.5 | 1 | 21 | 276 | 296 | GCAGCACCAUCAAGAUUCACA | UGGGAAUCUUGAUGAUGCUGC | Cleavage | [mRNA] locus=scaffold36:887280:890122:- |
| ssu-miR172 | willow_GLEAN_10004000 | 2.5 | 1 | 21 | 2136 | 2156 | GCAGCACCAUCAAGAUUCACA | UGUGAAUCCUGAUCGUGCUGU | Cleavage | [mRNA] locus=scaffold196:98172:101636:+ |
| ssu-miR172 | willow_GLEAN_10024912 | 3 | 1 | 21 | 759 | 780 | GCAGCAC-CAUCAAGAUUCACA | UGUGAUUCUUGAUGCGUGCUGU | Cleavage | [mRNA] locus=scaffold4:2977303:2980561:- |
| ssu-miR172 | willow_GLEAN_10020110 | 3 | 1 | 21 | 1170 | 1190 | GCAGCACCAUCAAGAUUCACA | UGUGGAUGUUGAUGCUGCUGC | Cleavage | [mRNA] locus=scaffold15:1640628:1644218:- |
| ssu-miR172 | willow_GLEAN_10007986 | 3 | 1 | 21 | 1161 | 1181 | GCAGCACCAUCAAGAUUCACA | UGUUAUUCUUGAUGGUGCUGG | Cleavage | [mRNA] locus=scaffold103:269109:273225:- |
| ssu-miR172 | willow_GLEAN_10020224 | 3 | 1 | 21 | 135 | 155 | GCAGCACCAUCAAGAUUCACA | UUUGAGUCUUGGCGGUGCUGC | Translation | [mRNA] locus=scaffold15:2445255:2447926:- |
| ssu-miR172 | willow_GLEAN_10025485 | 3 | 1 | 21 | 735 | 755 | GCAGCACCAUCAAGAUUCACA | AGUUAAUCAUGAUGGUGCUGG | Cleavage | [mRNA] locus=scaffold3:2357420:2363400:+ |
| ssu-miR396 | willow_GLEAN_10025670 | 2.5 | 1 | 21 | 345 | 366 | UUCCAC-AGCUUUCUUGAACUG | CCGUUCAAGAAAGCUUGUGGAA | Cleavage | [mRNA] locus=scaffold3:3702197:3705845:- |
| ssu-miR396 | willow_GLEAN_10008040 | 2.5 | 1 | 21 | 336 | 357 | UUCCACA-GCUUUCUUGAACUG | CCGUUCAAGAAAGCCUGUGGAA | Cleavage | [mRNA] locus=scaffold102:195891:197214:+ |
| ssu-miR396 | willow_GLEAN_10006541 | 2.5 | 1 | 21 | 399 | 420 | UUCCACA-GCUUUCUUGAACUG | CCGUUCAAGAAAGCCUGUGGAA | Cleavage | [mRNA] locus=scaffold127:138578:142928:- |
| ssu-miR396 | willow_GLEAN_10014833 | 2.5 | 1 | 21 | 345 | 366 | UUCCACA-GCUUUCUUGAACUG | CCGUUCAAGAAAGCCUGUGGAA | Cleavage | [mRNA] locus=scaffold37:983476:988361:- |
| ssu-miR396 | willow_GLEAN_10025311 | 2.5 | 1 | 21 | 717 | 738 | UUCCACA-GCUUUCUUGAACUG | CCGUUCAAGAAAGCCUGUGGAA | Cleavage | [mRNA] locus=scaffold3:1058177:1060283:- |
| ssu-miR396 | willow_GLEAN_10012353 | 2.5 | 1 | 21 | 573 | 594 | UUCCACA-GCUUUCUUGAACUG | CCGUUCAAGAAAGCAUGUGGAA | Cleavage | [mRNA] locus=scaffold51:966501:968325:+ |
| ssu-miR396 | willow_GLEAN_10023599 | 2.5 | 1 | 21 | 666 | 687 | UUCCACA-GCUUUCUUGAACUG | CCGUUCAAGAAAGCCUGUGGAA | Cleavage | [mRNA] locus=scaffold6:3397614:3399603:+ |
| ssu-miR396 | willow_GLEAN_10005202 | 2.5 | 1 | 21 | 648 | 669 | UUCCACA-GCUUUCUUGAACUG | CCGUUCAAGAAAGCAUGUGGAA | Cleavage | [mRNA] locus=scaffold160:346891:348919:+ |
| ssu-miR396 | willow_GLEAN_10014582 | 2.5 | 1 | 21 | 714 | 735 | UUCCACA-GCUUUCUUGAACUG | CCGUUCAAGAAAGCCUGUGGAA | Cleavage | [mRNA] locus=scaffold38:478314:483813:- |
| ssu-miR396 | willow_GLEAN_10020659 | 2.5 | 1 | 21 | 2628 | 2649 | UUCCACA-GCUUUCUUGAACUG | CCGUUCAAGAAAGCCUGUGGAA | Cleavage | [mRNA] locus=scaffold13:1445475:1459721:+ |
| ssu-miR396 | willow_GLEAN_10022060 | 2.5 | 1 | 21 | 327 | 348 | UUCCACA-GCUUUCUUGAACUG | UCGUUCAAGAAAGCCUGUGGAA | Cleavage | [mRNA] locus=scaffold9:2655337:2656538:- |
| ssu-miR396 | willow_GLEAN_10025554 | 2.5 | 1 | 21 | 1659 | 1679 | UUCCACAGCUUUCUUGAACUG | GAGAUUGAGAAAGUUGUGGAA | Cleavage | [mRNA] locus=scaffold3:2823795:2825525:- |
| ssu-miR396 | willow_GLEAN_10018904 | 2.5 | 1 | 21 | 883 | 903 | UUCCACAGCUUUCUUGAACUG | AAGGUGAAGGAAGCUGUGGAA | Cleavage | [mRNA] locus=scaffold19:1867051:1869084:- |
| ssu-miR396 | willow_GLEAN_10005448 | 3 | 1 | 21 | 595 | 615 | UUCCACAGCUUUCUUGAACUG | CAGUUCAAGAAAGCUUUGAAA | Cleavage | [mRNA] locus=scaffold153:93776:94402:+ |
| ssu-miR396 | willow_GLEAN_10021485 | 3 | 1 | 21 | 306 | 327 | UUCCACA-GCUUUCUUGAACUG | CAGUUCAGGCAAGCAUGUGGAA | Cleavage | [mRNA] locus=scaffold10:413657:415945:+ |
| ssu-miR396 | willow_GLEAN_10009454 | 3 | 1 | 21 | 429 | 450 | UUCCAC-AGCUUUCUUGAACUG | UAGUUUGAGAAAGUUUGUGGAA | Cleavage | [mRNA] locus=scaffold83:515684:517354:+ |
| ssu-miR396 | willow_GLEAN_10012253 | 3 | 1 | 21 | 1037 | 1057 | UUCCACAGCUUUCUUGAACUG | CAGUUUAUGGGAGUUGUGGAA | Cleavage | [mRNA] locus=scaffold51:229332:230531:+ |
| ssu-miR396 | willow_GLEAN_10022899 | 3 | 1 | 21 | 709 | 729 | UUCCACAGCUUUCUUGAACUG | CAGUUCAAGAGAGGUGUAGAA | Cleavage | [mRNA] locus=scaffold7:2347321:2349591:+ |
| ssu-miR396 | willow_GLEAN_10019848 | 3 | 1 | 21 | 1401 | 1421 | UUCCACAGCUUUCUUGAACUG | UUAUUCAAGAAAGUUGUGGGA | Cleavage | [mRNA] locus=scaffold17:1913720:1920948:+ |
| ssu-miR396 | willow_GLEAN_10014093 | 3 | 1 | 21 | 532 | 554 | UUCCACA-GCU-UUCUUGAACUG | GAGUUCAAGAACAGCCUGUGGAG | Translation | [mRNA] locus=scaffold41:726621:727528:- |
| ssu-miR396 | willow_GLEAN_10002936 | 3 | 1 | 21 | 949 | 971 | UUCC-ACAGCU-UUCUUGAACUG | GAGUUCAAGGACAGCUGUAGGAA | Translation | [mRNA] locus=scaffold247:133624:137735:+ |
| ssu-miR396 | willow_GLEAN_10013635 | 3 | 1 | 21 | 608 | 629 | UUCCACAGCUUUCUUGA-ACUG | UGGUGUUAGGGAAGCUGUGGAA | Cleavage | [mRNA] locus=scaffold44:872971:877631:- |
| ssu-miR396 | willow_GLEAN_10002316 | 3 | 1 | 21 | 2268 | 2289 | UUCCACAGC-UUUCUUGAACUG | CAGUGCAAGAAGUGUUGUGGAA | Translation | [mRNA] locus=scaffold342:36389:39319:- |
| ssu-miR396 | willow_GLEAN_10003310 | 3 | 1 | 21 | 3030 | 3051 | UUCCACAGC-UUUCUUGAACUG | CAGUGCAAGAAGUGUUGUGGAA | Translation | [mRNA] locus=scaffold222:6977:15273:- |
| ssu-miR396 | willow_GLEAN_10016502 | 3 | 1 | 21 | 922 | 942 | UUCCACAGCUUUCUUGAACUG | UACUUCAAGAAAGUUGUGGGU | Cleavage | [mRNA] locus=scaffold29:1596853:1598463:+ |
| ssu-miR396 | willow_GLEAN_10025920 | 3 | 1 | 21 | 871 | 891 | UUCCACAGCUUUCUUGAACUG | CAUUUGAAGAGAGCUGUGGAG | Cleavage | [mRNA] locus=scaffold2:1296990:1298081:- |
| ssu-miR396 | willow_GLEAN_10023177 | 3 | 1 | 21 | 364 | 384 | UUCCACAGCUUUCUUGAACUG | CAGCUCAAGGAAUCUGUGGAG | Translation | [mRNA] locus=scaffold6:397143:400520:- |
| ssu-miR396 | willow_GLEAN_10020998 | 3 | 1 | 21 | 1115 | 1135 | UUCCACAGCUUUCUUGAACUG | CUGCUCGAGGAAGCUGUGGAA | Cleavage | [mRNA] locus=scaffold12:1845250:1848897:+ |
| ssu-miR396 | willow_GLEAN_10003262 | 3 | 1 | 21 | 811 | 832 | UUCC-ACAGCUUUCUUGAACUG | GACUUCAAGAAAGUUGUUGGAA | Cleavage | [mRNA] locus=scaffold225:166335:170946:- |
| ssu-miR396 | willow_GLEAN_10022318 | 3 | 1 | 21 | 433 | 453 | UUCCACAGCUUUCUUGAACUG | GAGUUAGGGAAGGUUGUGGAA | Cleavage | [mRNA] locus=scaffold8:1198693:1201245:+ |
| ssu-miR396 | willow_GLEAN_10009038 | 3 | 1 | 21 | 458 | 478 | UUCCACAGCUUUCUUGAACUG | AAGAUCAGGGAGGCUGUGGGA | Cleavage | [mRNA] locus=scaffold88:685800:688606:- |
| ssu-miR396 | willow_GLEAN_10009721 | 3 | 1 | 21 | 763 | 783 | UUCCACAGCUUUCUUGAACUG | UAUUACAAGAGAGUUGUGGAA | Cleavage | [mRNA] locus=scaffold79:415195:416405:+ |
| ssu-miR396 | willow_GLEAN_10016069 | 3 | 1 | 21 | 838 | 858 | UUCCACAGCUUUCUUGAACUG | AAGCUCAAGCAAGCUGUGGAC | Cleavage | [mRNA] locus=scaffold30:262175:265474:+ |
| ssu-miR396 | willow_GLEAN_10022003 | 3 | 1 | 21 | 1639 | 1659 | UUCCACAGCUUUCUUGAACUG | GAGGUCAUGAAAGCUGUGGAU | Cleavage | [mRNA] locus=scaffold9:1947559:1953053:- |
| ssu-miR396 | willow_GLEAN_10016085 | 3 | 1 | 21 | 83 | 103 | UUCCACAGCUUUCUUGAACUG | AAAUUCAAGAAAUUUGUGGAG | Translation | [mRNA] locus=scaffold30:400621:400803:+ |
| ssu-miR472a | willow_GLEAN_10001511 | 1.5 | 1 | 22 | 691 | 712 | UUUUCCCAACUCCACCCAUCCC | GGUAUGGGUGGAGUUGGGAAGA | Cleavage | [mRNA] locus=scaffold01959:528:3584:+ |
| ssu-miR472a | willow_GLEAN_10001273 | 2.5 | 1 | 22 | 34 | 55 | UUUUCCCAACUCCACCCAUCCC | GGGAUGGGGGGAGUUGGUAAAA | Cleavage | [mRNA] locus=scaffold02970:384:2768:+ |
| ssu-miR472a | willow_GLEAN_10002033 | 2.5 | 1 | 22 | 394 | 415 | UUUUCCCAACUCCACCCAUCCC | GGGAUGGGGGGAGUUGGUAAAA | Cleavage | [mRNA] locus=scaffold533:8951:11734:- |
| ssu-miR472a | willow_GLEAN_10006738 | 2.5 | 1 | 22 | 1336 | 1357 | UUUUCCCAACUCCACCCAUCCC | GGGAUGGGGGGAGUUGGUAAAA | Cleavage | [mRNA] locus=scaffold124:444031:448771:+ |
| ssu-miR472a | willow_GLEAN_10002280 | 2.5 | 1 | 22 | 1594 | 1615 | UUUUCCCAACUCCACCCAUCCC | GGGAUGGGGGGAGUUGGUAAAA | Cleavage | [mRNA] locus=scaffold354:6417:11711:+ |
| ssu-miR472a | willow_GLEAN_10011429 | 2.5 | 1 | 22 | 23 | 44 | UUUUCCCAACUCCACCCAUCCC | UGUAUGGGUGGAGCUGGGAAGA | Translation | [mRNA] locus=scaffold60:492378:492689:+ |
| ssu-miR472a | willow_GLEAN_10018925 | 2.5 | 1 | 22 | 23 | 44 | UUUUCCCAACUCCACCCAUCCC | UGUAUGGGUGGAGCUGGGAAGA | Translation | [mRNA] locus=scaffold19:1999695:2000099:+ |
| ssu-miR472a | willow_GLEAN_10025599 | 2.5 | 1 | 22 | 2306 | 2328 | UUUUCCCAACUCCA-CCCAUCCC | CUGAUGAGAUGGGGUUGGGAAAA | Cleavage | [mRNA] locus=scaffold3:3201848:3213606:- |
| ssu-miR472a | willow_GLEAN_10002635 | 2.5 | 1 | 22 | 344 | 365 | UUUUCCCAACUCCACCCAUCCC | UUGAUGGGAGGAAUUGGGAAAG | Translation | [mRNA] locus=scaffold274:14446:14856:- |
| ssu-miR472a | willow_GLEAN_10006980 | 3 | 1 | 22 | 833 | 856 | UUUUCC-CAACUCCA-CCCAUCCC | UGGAUGGGAUGGAGUUGUGGAAGA | Cleavage | [mRNA] locus=scaffold120:231087:233513:- |
| ssu-miR472a | willow_GLEAN_10006870 | 3 | 1 | 22 | 592 | 613 | UUUUCCCAACUCCACCCAUCCC | GGGAUGGCUGGCCUUGGGAAAA | Translation | [mRNA] locus=scaffold122:508161:510983:+ |
| ssu-miR472a | willow_GLEAN_10005685 | 3 | 1 | 22 | 1273 | 1295 | UUUUC-CCAACUCCACCCAUCCC | UUGAUGGGUGGAGGUGGAGAAGA | Translation | [mRNA] locus=scaffold144:139410:140846:+ |
| ssu-miR472a | willow_GLEAN_10025076 | 3 | 1 | 22 | 485 | 507 | UUUUCCCAACUCCACC-CAUCCC | UCGAUGAGGAGGAGUUGGGGAAG | Cleavage | [mRNA] locus=scaffold4:4147918:4148601:+ |
| ssu-miR472a | willow_GLEAN_10010577 | 3 | 1 | 22 | 1181 | 1203 | UUUUCCCAACUCCA-CCCAUCCC | CUGAUGAGAUGGGGUUGGGGAAA | Cleavage | [mRNA] locus=scaffold69:38171:48101:- |
| ssu-miR472a | willow_GLEAN_10016751 | 3 | 1 | 22 | 33 | 54 | UUUUCCCAACUCCACCCAUCCC | AAGAUGGAUGGAUUUGGGAGGA | Translation | [mRNA] locus=scaffold28:1632236:1633556:- |
| ssu-miR472a | willow_GLEAN_10016755 | 3 | 1 | 22 | 282 | 303 | UUUUCCCAACUCCACCCAUCCC | AAGAUGGAUGGAUUUGGGAGGA | Translation | [mRNA] locus=scaffold28:1659911:1661447:- |
| ssu-miR472a | willow_GLEAN_10016753 | 3 | 1 | 22 | 636 | 657 | UUUUCCCAACUCCACCCAUCCC | AAGAUGGAUGGAUUUGGGAGGA | Translation | [mRNA] locus=scaffold28:1644138:1646215:- |
| ssu-miR472a | willow_GLEAN_10016754 | 3 | 1 | 22 | 741 | 762 | UUUUCCCAACUCCACCCAUCCC | AAGAUGGAUGGAUUUGGGAGGA | Translation | [mRNA] locus=scaffold28:1653526:1655716:- |
| ssu-miR472a | willow_GLEAN_10016752 | 3 | 1 | 22 | 741 | 762 | UUUUCCCAACUCCACCCAUCCC | AAGAUGGAUGGAUUUGGGAGGA | Translation | [mRNA] locus=scaffold28:1638063:1640081:- |
| ssu-miR472a | willow_GLEAN_10016757 | 3 | 1 | 22 | 738 | 759 | UUUUCCCAACUCCACCCAUCCC | AAGAUGGAUGGAUUUGGGAGGA | Translation | [mRNA] locus=scaffold28:1666893:1668916:- |
| ssu-miR472c | willow_GLEAN_10007470 | 2.5 | 1 | 22 | 645 | 666 | UCUUGCCUACUCCUCCCAUUCC | AGAAUGGCAGGAGAAGGCGAGA | Translation | [mRNA] locus=scaffold110:297428:299183:- |
| ssu-miR472c | willow_GLEAN_10020689 | 3 | 1 | 22 | 520 | 541 | UCUUGCCUACUCCUCCCAUUCC | GGAAUGGGGGGAGUCGGGAAGA | Cleavage | [mRNA] locus=scaffold13:1737662:1740340:- |
| ssu-miR472c | willow_GLEAN_10001441 | 3 | 1 | 22 | 56 | 77 | UCUUGCCUACUCCUCCCAUUCC | CGAAUGAGGGGAGUGGGCAAGC | Cleavage | [mRNA] locus=scaffold02193:314:3404:+ |
| ssu-miR472c | willow_GLEAN_10019227 | 3 | 1 | 22 | 485 | 506 | UCUUGCCUACUCCUCCCAUUCC | GUUCUGGGAAGAGUAGGCAAGA | Cleavage | [mRNA] locus=scaffold18:2029918:2032218:- |
| ssu-miR6423 | willow_GLEAN_10023371 | 2.5 | 1 | 22 | 599 | 620 | CCGCUGUCGCCACUAUCUUCCU | GGGAAGAGAGUGGCGGCGGUGG | Cleavage | [mRNA] locus=scaffold6:1708359:1709476:- |
| ssu-miR6423 | willow_GLEAN_10019839 | 3 | 1 | 22 | 10 | 31 | CCGCUGUCGCCACUAUCUUCCU | AGGAAAAUAGUGGUGGUAGUGG | Cleavage | [mRNA] locus=scaffold17:1835591:1837775:- |
| ssu-miR6423 | willow_GLEAN_10008600 | 3 | 1 | 22 | 169 | 190 | CCGCUGUCGCCACUAUCUUCCU | AGGAAGAUGGUGGAGAUGGUGG | Translation | [mRNA] locus=scaffold94:543702:544209:+ |
| ssu-miR6423 | willow_GLEAN_10023982 | 3 | 1 | 22 | 652 | 673 | CCGCUGUCGCCACUAUCUUCCU | CGGAGGACGGUGGUGACAGUGG | Cleavage | [mRNA] locus=scaffold5:1227923:1229754:- |
| ssu-miR7841 | willow_GLEAN_10026664 | 2 | 1 | 21 | 329 | 349 | GGGGGUUGCUGUCAAGCAUAA | GUGUGUUUGAUAGCAACCCCU | Cleavage | [mRNA] locus=scaffold1:1268705:1269742:- |
| ssu-miR7841 | willow_GLEAN_10020679 | 3 | 1 | 21 | 2416 | 2436 | GGGGGUUGCUGUCAAGCAUAA | CUAUGUUUGUCAGCAAUCUCU | Cleavage | [mRNA] locus=scaffold13:1621602:1634247:- |
| ssu-miR7841 | willow_GLEAN_10000878 | 3 | 1 | 21 | 478 | 498 | GGGGGUUGCUGUCAAGCAUAA | CUAGGCAUGACAGCAACCUUC | Cleavage | [mRNA] locus=scaffold05419:736:2121:- |
| ssu-miR7841 | willow_GLEAN_10024915 | 3 | 1 | 21 | 1178 | 1198 | GGGGGUUGCUGUCAAGCAUAA | AUAUGCUUGCCAACAACCUUC | Translation | [mRNA] locus=scaffold4:2988780:2993856:+ |
| ssu-miR7841 | willow_GLEAN_10020084 | 3 | 1 | 21 | 1246 | 1266 | GGGGGUUGCUGUCAAGCAUAA | GUAUACUUGUCAGCAGCCCUC | Cleavage | [mRNA] locus=scaffold15:1460424:1464200:- |
| ssu-miR7841 | willow_GLEAN_10014755 | 3 | 1 | 21 | 1718 | 1738 | GGGGGUUGCUGUCAAGCAUAA | CUAUUCUUGACAGAAAUCCUC | Cleavage | [mRNA] locus=scaffold37:402007:410173:+ |
