# Supplementary material for: Detecting the Candidate Gender Determinants by Bioinformatic Prediction of miRNAs and Their Targets from Transcriptome Sequences of the Male and Female Flowers in Salix suchowensis
Source: Biomed Res Int. 2017 May 30;2017:9614596. doi: 10.1155/2017/9614596 (PMC5468582; doi:10.1155/2017/9614596)
Supplement: Supplementary file 1 — Figure S1: Number of targets for each miRNA in S. suchowensis. Green bars represent miRNAs common to female and male, whereas the red and blue bars correspond to miRNAs present exclusively in female or male, respectively. Figure S2: Compare the number targets in different component groups between female and male flower buds of S. suchowensis. Figure S3: Comparison of the expression levels of miRNA target genes between male and female buds of S. suchowensis. Color scales represent TPM normalized log2 transformed counts, whereas the red scales indicate high expression, and the blue scales indicate low expression. Table S1: Predicted miRNA target genes in female flower buds of S. suchowensis. Table S2: Predicted miRNA target genes in male flower buds of S. suchowensis. Table S3: GO annotation of miRNA targets identified in female flower buds of S. suchowensis. Table S4: GO annotation of miRNA targets identified in male flower buds of S. suchowensis. Table S5: Different expression of miRNA targets between female and male flower buds of S. suchowensis. Table S6: Distribution of miRNA target genes on 19 chromosomes of S. suchowensis. [file 9614596.f1.zip › Supplementary Materials/Table S3.doc]

***Table S3.*** *GO annotation of miRNA targets identified in female flower buds of S. suchowensis.*

| **miRNA** | **Target gene** | **Gene Ontology (GO) annotation of target genes** |
| --- | --- | --- |
| ssu-miR472a | willow_GLEAN_10001273 | GO:0005524; ATP binding; Molecular Function  GO:0006915; apoptosis; Biological Process  GO:0006952; defense response; Biological Process |
| ssu-miR472a | willow_GLEAN_10001511 | GO:0005524; ATP binding; Molecular Function  GO:0006915; apoptosis; Biological Process  GO:0006952; defense response; Biological Process |
| ssu-miR472a | willow_GLEAN_10002033 | GO:0005515; protein binding; Molecular Function  GO:0005524; ATP binding; Molecular Function  GO:0006915; apoptosis; Biological Process  GO:0006952; defense response; Biological Process |
| ssu-miR472a | willow_GLEAN_10002280 | GO:0005524; ATP binding; Molecular Function  GO:0006915; apoptosis; Biological Process  GO:0006952; defense response; Biological Process |
| ssu-miR390 | willow_GLEAN_10002294 | GO:0004506; squalene monooxygenase activity; Molecular Function  GO:0016021; integral to membrane; Cellular Component  GO:0050660; FAD or FADH2 binding; Molecular Function  GO:0055114; oxidation-reduction process; Biological Process |
| ssu-miR1448 | willow_GLEAN_10002295 | GO:0003735; structural constituent of ribosome; Molecular Function  GO:0005622; intracellular; Cellular Component  GO:0005840; ribosome; Cellular Component  GO:0006412; translation; Biological Process |
| ssu-miR166 | willow_GLEAN_10002393 | GO:0009244; lipopolysaccharide core region biosynthetic process; Biological Process |
| ssu-miR472a | willow_GLEAN_10002635 | GO:0008152; metabolic process; Biological Process  GO:0016846; carbon-sulfur lyase activity; Molecular Function |
| ssu-miR1448 | willow_GLEAN_10003110 | GO:0005524; ATP binding; Molecular Function  GO:0006915; apoptosis; Biological Process  GO:0006952; defense response; Biological Process |
| ssu-miR166 | willow_GLEAN_10004638 | GO:0009244; lipopolysaccharide core region biosynthetic process; Biological Process |
| ssu-miR472a | willow_GLEAN_10005685 | GO:0008152; metabolic process; Biological Process  GO:0016758; transferase activity, transferring hexosyl groups; Molecular Function |
| ssu-miR172a | willow_GLEAN_10006512 | GO:0003700; sequence-specific DNA binding transcription factor activity; Molecular Function  GO:0006355; regulation of transcription, DNA-dependent; Biological Process |
| ssu-miR472a | willow_GLEAN_10006738 | GO:0005524; ATP binding; Molecular Function  GO:0006915; apoptosis; Biological Process  GO:0006952; defense response; Biological Process |
| ssu-miR172a | willow_GLEAN_10006848 | GO:0004221; ubiquitin thiolesterase activity; Molecular Function  GO:0006511; ubiquitin-dependent protein catabolic process; Biological Process  GO:0008270; zinc ion binding; Molecular Function |
| ssu-miR472a | willow_GLEAN_10006870 | GO:0005524; ATP binding; Molecular Function  GO:0006915; apoptosis; Biological Process  GO:0006952; defense response; Biological Process |
| ssu-miR172a | willow_GLEAN_10007066 | GO:0005509; calcium ion binding; Molecular Function  GO:0005544; calcium-dependent phospholipid binding; Molecular Function |
| ssu-miR172a | willow_GLEAN_10008165 | GO:0003700; sequence-specific DNA binding transcription factor activity; Molecular Function  GO:0006355; regulation of transcription, DNA-dependent; Biological Process |
| ssu-miR1448 | willow_GLEAN_10008484 | GO:0005524; ATP binding; Molecular Function  GO:0006915; apoptosis; Biological Process  GO:0006952; defense response; Biological Process |
| ssu-miR162 | willow_GLEAN_10009152 | GO:0005524; ATP binding; Molecular Function  GO:0006457; protein folding; Biological Process  GO:0051082; unfolded protein binding; Molecular Function |
| ssu-miR169 | willow_GLEAN_10009255 | GO:0003924; GTPase activity; Molecular Function  GO:0005525; GTP binding; Molecular Function |
| ssu-miR472a | willow_GLEAN_10010577 | GO:0003676; nucleic acid binding; Molecular Function  GO:0003677; DNA binding; Molecular Function  GO:0004386; helicase activity; Molecular Function  GO:0005524; ATP binding; Molecular Function |
| ssu-miR172a | willow_GLEAN_10010690 | GO:0003700; sequence-specific DNA binding transcription factor activity; Molecular Function  GO:0006355; regulation of transcription, DNA-dependent; Biological Process |
| ssu-miR472a | willow_GLEAN_10011429 | GO:0003677; DNA binding; Molecular Function |
| ssu-miR166 | willow_GLEAN_10011433 | GO:0005524; ATP binding; Molecular Function  GO:0006810; transport; Biological Process  GO:0016021; integral to membrane; Cellular Component  GO:0016887; ATPase activity; Molecular Function  GO:0042626; ATPase activity, coupled to transmembrane movement of substances; Molecular Function  GO:0055085; transmembrane transport; Biological Process  GO:0000166; nucleotide binding; Molecular Function  GO:0017111; nucleoside-triphosphatase activity; Molecular Function |
| ssu-miR172a | willow_GLEAN_10015032 | GO:0004672; protein kinase activity; Molecular Function  GO:0004674; protein serine/threonine kinase activity; Molecular Function  GO:0005524; ATP binding; Molecular Function  GO:0006468; protein phosphorylation; Biological Process |
| ssu-miR472a | willow_GLEAN_10016751 | GO:0004497; monooxygenase activity; Molecular Function  GO:0005506; iron ion binding; Molecular Function  GO:0009055; electron carrier activity; Molecular Function  GO:0020037; heme binding; Molecular Function  GO:0055114; oxidation-reduction process; Biological Process |
| ssu-miR472a | willow_GLEAN_10016752 | GO:0004497; monooxygenase activity; Molecular Function  GO:0005506; iron ion binding; Molecular Function  GO:0009055; electron carrier activity; Molecular Function  GO:0020037; heme binding; Molecular Function |
| ssu-miR472a | willow_GLEAN_10016753 | GO:0004497; monooxygenase activity; Molecular Function  GO:0005506; iron ion binding; Molecular Function  GO:0009055; electron carrier activity; Molecular Function  GO:0020037; heme binding; Molecular Function  GO:0055114; oxidation-reduction process; Biological Process |
| ssu-miR472a | willow_GLEAN_10016754 | GO:0004497; monooxygenase activity; Molecular Function  GO:0005506; iron ion binding; Molecular Function  GO:0009055; electron carrier activity; Molecular Function  GO:0020037; heme binding; Molecular Function  GO:0055114; oxidation-reduction process; Biological Process |
| ssu-miR472a | willow_GLEAN_10016755 | GO:0004497; monooxygenase activity; Molecular Function  GO:0005506; iron ion binding; Molecular Function  GO:0009055; electron carrier activity; Molecular Function  GO:0020037; heme binding; Molecular Function  GO:0055114; oxidation-reduction process; Biological Process |
| ssu-miR472a | willow_GLEAN_10016757 | GO:0004497; monooxygenase activity; Molecular Function  GO:0005506; iron ion binding; Molecular Function  GO:0009055; electron carrier activity; Molecular Function  GO:0020037; heme binding; Molecular Function  GO:0055114; oxidation-reduction process; Biological Process |
| ssu-miR1448 | willow_GLEAN_10017442 | GO:0000166; nucleotide binding; Molecular Function  GO:0005515; protein binding; Molecular Function  GO:0005524; ATP binding; Molecular Function  GO:0006810; transport; Biological Process  GO:0016021; integral to membrane; Cellular Component  GO:0016887; ATPase activity; Molecular Function  GO:0042626; ATPase activity, coupled to transmembrane movement of substances; Molecular Function  GO:0055085; transmembrane transport; Biological Process |
| ssu-miR172a | willow_GLEAN_10018050 | GO:0004396; hexokinase activity; Molecular Function  GO:0005524; ATP binding; Molecular Function  GO:0005975; carbohydrate metabolic process; Biological Process  GO:0006096; glycolysis; Biological Process  GO:0016773; phosphotransferase activity, alcohol group as acceptor; Molecular Function |
| ssu-miR472a | willow_GLEAN_10018925 | GO:0003677; DNA binding; Molecular Function |
| ssu-miR172a | willow_GLEAN_10019574 | GO:0003700; sequence-specific DNA binding transcription factor activity; Molecular Function  GO:0006355; regulation of transcription, DNA-dependent; Biological Process |
| ssu-miR172a | willow_GLEAN_10019637 | GO:0005515; protein binding; Molecular Function  GO:0005622; intracellular; Cellular Component  GO:0008270; zinc ion binding; Molecular Function |
| ssu-miR6423 | willow_GLEAN_10019839 | GO:0006950; response to stress; Biological Process |
| ssu-miR172a | willow_GLEAN_10020581 | GO:0003700; sequence-specific DNA binding transcription factor activity; Molecular Function  GO:0006355; regulation of transcription, DNA-dependent; Biological Process |
| ssu-miR162 | willow_GLEAN_10021194 | GO:0004672; protein kinase activity; Molecular Function  GO:0004674; protein serine/threonine kinase activity; Molecular Function  GO:0005524; ATP binding; Molecular Function  GO:0006468; protein phosphorylation; Biological Process |
| ssu-miR172a | willow_GLEAN_10021209 | GO:0006284; base-excision repair; Biological Process  GO:0008725; DNA-3-methyladenine glycosylase I activity; Molecular Function |
| ssu-miR172a | willow_GLEAN_10024780 | GO:0003700; sequence-specific DNA binding transcription factor activity; Molecular Function  GO:0006355; regulation of transcription, DNA-dependent; Biological Process |
| ssu-miR472a | willow_GLEAN_10025076 | GO:0005515; protein binding; Molecular Function  GO:0008270; zinc ion binding; Molecular Function |
| ssu-miR472a | willow_GLEAN_10025599 | GO:0000785; chromatin; Cellular Component  GO:0003676; nucleic acid binding; Molecular Function  GO:0003677; DNA binding; Molecular Function  GO:0003682; chromatin binding; Molecular Function  GO:0004386; helicase activity; Molecular Function  GO:0005515; protein binding; Molecular Function  GO:0005524; ATP binding; Molecular Function  GO:0005634; nucleus; Cellular Component  GO:0006333; chromatin assembly or disassembly; Biological Process  GO:0008270; zinc ion binding; Molecular Function |
| ssu-miR166 | willow_GLEAN_10026446 | GO:0019538; protein metabolic process; Biological Process GO:0005524; ATP binding; Molecular Function  GO:0005737; cytoplasm; Cellular Component  GO:0009408; response to heat; Biological Process  GO:0016485; protein processing; Biological Process  GO:0000166; nucleotide binding; Molecular Function  GO:0017111; nucleoside-triphosphatase activity; Molecular Function |
