# Supplementary material for: Detecting the Candidate Gender Determinants by Bioinformatic Prediction of miRNAs and Their Targets from Transcriptome Sequences of the Male and Female Flowers in Salix suchowensis
Source: Biomed Res Int. 2017 May 30;2017:9614596. doi: 10.1155/2017/9614596 (PMC5468582; doi:10.1155/2017/9614596)
Supplement: Supplementary file 1 — Figure S1: Number of targets for each miRNA in S. suchowensis. Green bars represent miRNAs common to female and male, whereas the red and blue bars correspond to miRNAs present exclusively in female or male, respectively. Figure S2: Compare the number targets in different component groups between female and male flower buds of S. suchowensis. Figure S3: Comparison of the expression levels of miRNA target genes between male and female buds of S. suchowensis. Color scales represent TPM normalized log2 transformed counts, whereas the red scales indicate high expression, and the blue scales indicate low expression. Table S1: Predicted miRNA target genes in female flower buds of S. suchowensis. Table S2: Predicted miRNA target genes in male flower buds of S. suchowensis. Table S3: GO annotation of miRNA targets identified in female flower buds of S. suchowensis. Table S4: GO annotation of miRNA targets identified in male flower buds of S. suchowensis. Table S5: Different expression of miRNA targets between female and male flower buds of S. suchowensis. Table S6: Distribution of miRNA target genes on 19 chromosomes of S. suchowensis. [file 9614596.f1.zip › Supplementary Materials/Table S4.doc]

**Table S4.** GO annotation of miRNA targets identified in male flower buds of *S. suchowensis.*

| **miRNAs** | **Target gene** | **Gene Ontology (GO) annotation of target genes** |
| --- | --- | --- |
| ssu-miR7841 | willow_GLEAN_10000878 | GO:0016020; membrane; Cellular Component  GO:0016798; hydrolase activity, acting on glycosyl bonds; Molecular Function |
| ssu-miR472a | willow_GLEAN_10001273 | GO:0005524; ATP binding; Molecular Function  GO:0006915; apoptosis; Biological Process  GO:0006952; defense response; Biological Process |
| ssu-miR472c | willow_GLEAN_10001441 | GO:0016491; oxidoreductase activity; Molecular Function  GO:0055114; oxidation-reduction process; Biological Process |
| ssu-miR472a | willow_GLEAN_10001511 | GO:0005524; ATP binding; Molecular Function  GO:0006915; apoptosis; Biological Process  GO:0006952; defense response; Biological Process |
| ssu-miR472a | willow_GLEAN_10002033 | GO:0005515; protein binding; Molecular Function  GO:0005524; ATP binding; Molecular Function  GO:0006915; apoptosis; Biological Process  GO:0006952; defense response; Biological Process |
| ssu-miR472a | willow_GLEAN_10002280 | GO:0005524; ATP binding; Molecular Function  GO:0006915; apoptosis; Biological Process  GO:0006952; defense response; Biological Process |
| ssu-miR1448 | willow_GLEAN_10002295 | GO:0003735; structural constituent of ribosome; Molecular Function  GO:0005622; intracellular; Cellular Component  GO:0005840; ribosome; Cellular Component  GO:0006412; translation; Biological Process |
| ssu-miR396 | willow_GLEAN_10002316 | GO:0004672; protein kinase activity; Molecular Function  GO:0005524; ATP binding; Molecular Function  GO:0005529; sugar binding; Molecular Function  GO:0006468; protein phosphorylation; Biological Process |
| ssu-miR472a | willow_GLEAN_10002635 | GO:0008152; metabolic process; Biological Process  GO:0016846; carbon-sulfur lyase activity; Molecular Function |
| ssu-miR156 | willow_GLEAN_10002818 | GO:0003677; DNA binding; Molecular Function  GO:0005634; nucleus; Cellular Component |
| ssu-miR1448 | willow_GLEAN_10003110 | GO:0005524; ATP binding; Molecular Function  GO:0006915; apoptosis; Biological Process  GO:0006952; defense response; Biological Process |
| ssu-miR396 | willow_GLEAN_10003262 | GO:0008219; cell death; Biological Process  GO:0016021; integral to membrane; Cellular Component |
| ssu-miR396 | willow_GLEAN_10003310 | GO:0004672; protein kinase activity; Molecular Function  GO:0005524; ATP binding; Molecular Function  GO:0005529; sugar binding; Molecular Function  GO:0006468; protein phosphorylation; Biological Process |
| ssu-miR172b/c | willow_GLEAN_10004000 | GO:0004252; serine-type endopeptidase activity; Molecular Function  GO:0006508; proteolysis; Biological Process  GO:0042802; identical protein binding; Molecular Function  GO:0043086; negative regulation of catalytic activity; Biological Process |
| ssu-miR156 | willow_GLEAN_10004254 | GO:0000159; protein phosphatase type 2A complex; Cellular Component  GO:0005515; protein binding; Molecular Function  GO:0007165; signal transduction; Biological Process  GO:0008601; protein phosphatase type 2A regulator activity; Molecular Function |
| ssu-miR396 | willow_GLEAN_10005202 | GO:0005515; protein binding; Molecular Function  GO:0005524; ATP binding; Molecular Function  GO:0005634; nucleus; Cellular Component  GO:0016818; hydrolase activity, acting on acid anhydrides, in phosphorus-containing anhydrides; Molecular Function  GO:0045449; regulation of transcription; Biological Process |
| ssu-miR396 | willow_GLEAN_10005448 | GO:0004866; endopeptidase inhibitor activity; Molecular Function |
| ssu-miR472a | willow_GLEAN_10005685 | GO:0008152; metabolic process; Biological Process  GO:0016758; transferase activity, transferring hexosyl groups; Molecular Function |
| ssu-miR156 | willow_GLEAN_10006436 | GO:0005643; nuclear pore; Cellular Component  GO:0006810; transport; Biological Process |
| ssu-miR396 | willow_GLEAN_10006541 | GO:0005515; protein binding; Molecular Function  GO:0005524; ATP binding; Molecular Function  GO:0005634; nucleus; Cellular Component  GO:0016818; hydrolase activity, acting on acid anhydrides, in phosphorus-containing anhydrides; Molecular Function  GO:0045449; regulation of transcription; Biological Process |
| ssu-miR472a | willow_GLEAN_10006738 | GO:0005524; ATP binding; Molecular Function  GO:0006915; apoptosis; Biological Process  GO:0006952; defense response; Biological Process |
| ssu-miR472a | willow_GLEAN_10006870 | GO:0005524; ATP binding; Molecular Function  GO:0006915; apoptosis; Biological Process  GO:0006952; defense response; Biological Process |
| ssu-miR472c | willow_GLEAN_10007470 | GO:0004497; monooxygenase activity; Molecular Function  GO:0005506; iron ion binding; Molecular Function  GO:0009055; electron carrier activity; Molecular Function  GO:0020037; heme binding; Molecular Function  GO:0055114; oxidation-reduction process; Biological Process |
| ssu-miR172b/c | willow_GLEAN_10007986 | GO:0005524; ATP binding; Molecular Function  GO:0005737; cytoplasm; Cellular Component  GO:0006457; protein folding; Biological Process  GO:0044267; cellular protein metabolic process; Biological Process  GO:0042026; protein refolding; Biological Process |
| ssu-miR156 | willow_GLEAN_10007998 | GO:0003677; DNA binding; Molecular Function  GO:0005634; nucleus; Cellular Component |
| ssu-miR156 | willow_GLEAN_10008019 | GO:0003677; DNA binding; Molecular Function  GO:0003896; DNA primase activity; Molecular Function  GO:0005634; nucleus; Cellular Component  GO:0006269; DNA replication, synthesis of RNA primer; Biological Process |
| ssu-miR396 | willow_GLEAN_10008040 | GO:0005515; protein binding; Molecular Function  GO:0005524; ATP binding; Molecular Function  GO:0005634; nucleus; Cellular Component  GO:0016818; hydrolase activity, acting on acid anhydrides, in phosphorus-containing anhydrides; Molecular Function  GO:0045449; regulation of transcription; Biological Process |
| ssu-miR1448 | willow_GLEAN_10008484 | GO:0005524; ATP binding; Molecular Function  GO:0006915; apoptosis; Biological Process  GO:0006952; defense response; Biological Process |
| ssu-miR396 | willow_GLEAN_10009038 | GO:0004197; cysteine-type endopeptidase activity; Molecular Function  GO:0006508; proteolysis; Biological Process  GO:0008234; cysteine-type peptidase activity; Molecular Function |
| ssu-miR162 | willow_GLEAN_10009152 | GO:0005524; ATP binding; Molecular Function  GO:0006457; protein folding; Biological Process  GO:0051082; unfolded protein binding; Molecular Function |
| ssu-miR169 | willow_GLEAN_10009255 | GO:0003924; GTPase activity; Molecular Function  GO:0005525; GTP binding; Molecular Function |
| ssu-miR396 | willow_GLEAN_10009721 | GO:0004601; peroxidase activity; Molecular Function  GO:0006979; response to oxidative stress; Biological Process  GO:0020037; heme binding; Molecular Function  GO:0055114; oxidation-reduction process; Biological Process |
| ssu-miR160 | willow_GLEAN_10010173 | GO:0003677; DNA binding; Molecular Function  GO:0005634; nucleus; Cellular Component  GO:0006355; regulation of transcription, DNA-dependent; Biological Process  GO:0009725; response to hormone stimulus; Biological Process  GO:0045449; regulation of transcription; Biological Process  GO:0046983; protein dimerization activity; Molecular Function |
| ssu-miR156 | willow_GLEAN_10010327 | GO:0003677; DNA binding; Molecular Function  GO:0005634; nucleus; Cellular Component |
| ssu-miR167 | willow_GLEAN_10010398 | GO:0016491; oxidoreductase activity; Molecular Function  GO:0055114; oxidation-reduction process; Biological Process |
| ssu-miR472a | willow_GLEAN_10010577 | GO:0003676; nucleic acid binding; Molecular Function  GO:0003677; DNA binding; Molecular Function  GO:0004386; helicase activity; Molecular Function  GO:0005524; ATP binding; Molecular Function |
| ssu-miR167 | willow_GLEAN_10011083 | GO:0016491; oxidoreductase activity; Molecular Function  GO:0055114; oxidation-reduction process; Biological Process |
| ssu-miR156 | willow_GLEAN_10011152 | GO:0003677; DNA binding; Molecular Function  GO:0005634; nucleus; Cellular Component |
| ssu-miR472a | willow_GLEAN_10011429 | GO:0003677; DNA binding; Molecular Function |
| ssu-miR156 | willow_GLEAN_10011703 | GO:0003677; DNA binding; Molecular Function  GO:0005634; nucleus; Cellular Component |
| ssu-miR160 | willow_GLEAN_10012047 | GO:0003677; DNA binding; Molecular Function  GO:0005634; nucleus; Cellular Component  GO:0006355; regulation of transcription, DNA-dependent; Biological Process  GO:0009725; response to hormone stimulus; Biological Process  GO:0045449; regulation of transcription; Biological Process |
| ssu-miR396 | willow_GLEAN_10012353 | GO:0005515; protein binding; Molecular Function  GO:0005524; ATP binding; Molecular Function  GO:0005634; nucleus; Cellular Component  GO:0016818; hydrolase activity, acting on acid anhydrides, in phosphorus-containing anhydrides; Molecular Function  GO:0045449; regulation of transcription; Biological Process |
| ssu-miR156 | willow_GLEAN_10012461 | GO:0006865; amino acid transport; Biological Process  GO:0016021; integral to membrane; Cellular Component GO:0006810; transport; Biological Process  GO:0016020; membrane; Cellular Component  GO:0055085; transmembrane transport; Biological Process  GO:0015171; amino acid transmembrane transporter activity; Molecular Function |
| ssu-miR160 | willow_GLEAN_10013064 | GO:0003677; DNA binding; Molecular Function  GO:0005634; nucleus; Cellular Component  GO:0006355; regulation of transcription, DNA-dependent; Biological Process  GO:0009725; response to hormone stimulus; Biological Process  GO:0045449; regulation of transcription; Biological Process  GO:0046983; protein dimerization activity; Molecular Function |
| ssu-miR156 | willow_GLEAN_10013536 | GO:0003677; DNA binding; Molecular Function  GO:0005634; nucleus; Cellular Component |
| ssu-miR396 | willow_GLEAN_10013635 | GO:0005515; protein binding; Molecular Function |
| ssu-miR396 | willow_GLEAN_10014582 | GO:0005515; protein binding; Molecular Function  GO:0005524; ATP binding; Molecular Function  GO:0005634; nucleus; Cellular Component  GO:0016818; hydrolase activity, acting on acid anhydrides, in phosphorus-containing anhydrides; Molecular Function  GO:0045449; regulation of transcription; Biological Process |
| ssu-miR7841 | willow_GLEAN_10014755 | GO:0005515; protein binding; Molecular Function |
| ssu-miR396 | willow_GLEAN_10014833 | GO:0005515; protein binding; Molecular Function  GO:0005524; ATP binding; Molecular Function  GO:0005634; nucleus; Cellular Component  GO:0016818; hydrolase activity, acting on acid anhydrides, in phosphorus-containing anhydrides; Molecular Function  GO:0045449; regulation of transcription; Biological Process |
| ssu-miR396 | willow_GLEAN_10016069 | GO:0000154; rRNA modification; Biological Process  GO:0000179; rRNA (adenine-N6,N6-)-dimethyltransferase activity; Molecular Function  GO:0008649; rRNA methyltransferase activity; Molecular Function |
| ssu-miR156 | willow_GLEAN_10016441 | GO:0003677; DNA binding; Molecular Function  GO:0005634; nucleus; Cellular Component |
| ssu-miR396 | willow_GLEAN_10016502 | GO:0005215; transporter activity; Molecular Function  GO:0006810; transport; Biological Process  GO:0016020; membrane; Cellular Component  GO:0055085; transmembrane transport; Biological Process |
| ssu-miR156 | willow_GLEAN_10016624 | GO:0003677; DNA binding; Molecular Function  GO:0005634; nucleus; Cellular Component |
| ssu-miR472a | willow_GLEAN_10016751 | GO:0004497; monooxygenase activity; Molecular Function  GO:0005506; iron ion binding; Molecular Function  GO:0009055; electron carrier activity; Molecular Function  GO:0020037; heme binding; Molecular Function  GO:0055114; oxidation-reduction process; Biological Process |
| ssu-miR472a | willow_GLEAN_10016752 | GO:0004497; monooxygenase activity; Molecular Function  GO:0005506; iron ion binding; Molecular Function  GO:0009055; electron carrier activity; Molecular Function  GO:0020037; heme binding; Molecular Function  GO:0055114; oxidation-reduction process; Biological Process |
| ssu-miR472a | willow_GLEAN_10016753 | GO:0004497; monooxygenase activity; Molecular Function  GO:0005506; iron ion binding; Molecular Function  GO:0009055; electron carrier activity; Molecular Function  GO:0020037; heme binding; Molecular Function  GO:0055114; oxidation-reduction process; Biological Process |
| ssu-miR472a | willow_GLEAN_10016754 | GO:0004497; monooxygenase activity; Molecular Function  GO:0005506; iron ion binding; Molecular Function  GO:0009055; electron carrier activity; Molecular Function  GO:0020037; heme binding; Molecular Function  GO:0055114; oxidation-reduction process; Biological Process |
| ssu-miR472a | willow_GLEAN_10016755 | GO:0004497; monooxygenase activity; Molecular Function  GO:0005506; iron ion binding; Molecular Function  GO:0009055; electron carrier activity; Molecular Function  GO:0020037; heme binding; Molecular Function  GO:0055114; oxidation-reduction process; Biological Process |
| ssu-miR472a | willow_GLEAN_10016757 | GO:0004497; monooxygenase activity; Molecular Function  GO:0005506; iron ion binding; Molecular Function  GO:0009055; electron carrier activity; Molecular Function  GO:0020037; heme binding; Molecular Function  GO:0055114; oxidation-reduction process; Biological Process |
| ssu-miR1448 | willow_GLEAN_10017442 | GO:0000166; nucleotide binding; Molecular Function  GO:0005515; protein binding; Molecular Function  GO:0005524; ATP binding; Molecular Function  GO:0006810; transport; Biological Process  GO:0016021; integral to membrane; Cellular Component  GO:0016887; ATPase activity; Molecular Function  GO:0042626; ATPase activity, coupled to transmembrane movement of substances; Molecular Function  GO:0055085; transmembrane transport; Biological Process  GO:0017111; nucleoside-triphosphatase activity; Molecular Function |
| ssu-miR472a | willow_GLEAN_10018925 | GO:0003677; DNA binding; Molecular Function |
| ssu-miR472c | willow_GLEAN_10019227 | GO:0005622; intracellular; Cellular Component  GO:0008270; zinc ion binding; Molecular Function |
| ssu-miR160 | willow_GLEAN_10019627 | GO:0003677; DNA binding; Molecular Function  GO:0005634; nucleus; Cellular Component  GO:0006355; regulation of transcription, DNA-dependent; Biological Process  GO:0009725; response to hormone stimulus; Biological Process  GO:0045449; regulation of transcription; Biological Process  GO:0046983; protein dimerization activity; Molecular Function |
| ssu-miR6423 | willow_GLEAN_10019839 | GO:0006950; response to stress; Biological Process |
| ssu-miR396 | willow_GLEAN_10019848 | GO:0003723; RNA binding; Molecular Function  GO:0006396; RNA processing; Biological Process |
| ssu-miR7841 | willow_GLEAN_10020084 | GO:0006813; potassium ion transport; Biological Process  GO:0015079; potassium ion transmembrane transporter activity; Molecular Function  GO:0016020; membrane; Cellular Component |
| ssu-miR172b/c | willow_GLEAN_10020110 | GO:0005525; GTP binding; Molecular Function  GO:0005622; intracellular; Cellular Component |
| ssu-miR172b/c | willow_GLEAN_10020224 | GO:0046983; protein dimerization activity; Molecular Function  GO:0005634; nucleus; Cellular Component  GO:0045449; regulation of transcription; Biological Process |
| ssu-miR396 | willow_GLEAN_10020659 | GO:0005515; protein binding; Molecular Function  GO:0005524; ATP binding; Molecular Function  GO:0005634; nucleus; Cellular Component  GO:0016818; hydrolase activity, acting on acid anhydrides, in phosphorus-containing anhydrides; Molecular Function  GO:0045449; regulation of transcription; Biological Process |
| ssu-miR7841 | willow_GLEAN_10020679 | GO:0003774; motor activity; Molecular Function  GO:0005515; protein binding; Molecular Function  GO:0005524; ATP binding; Molecular Function  GO:0016459; myosin complex; Cellular Component |
| ssu-miR472c | willow_GLEAN_10020689 | GO:0005515; protein binding; Molecular Function  GO:0005524; ATP binding; Molecular Function  GO:0006915; apoptosis; Biological Process  GO:0006952; defense response; Biological Process  GO:0000166; nucleotide binding; Molecular Function  GO:0017111; nucleoside-triphosphatase activity; Molecular Function |
| ssu-miR160 | willow_GLEAN_10020883 | GO:0003677; DNA binding; Molecular Function  GO:0005634; nucleus; Cellular Component  GO:0006355; regulation of transcription, DNA-dependent; Biological Process  GO:0009725; response to hormone stimulus; Biological Process  GO:0045449; regulation of transcription; Biological Process |
| ssu-miR162 | willow_GLEAN_10021194 | GO:0004672; protein kinase activity; Molecular Function  GO:0004674; protein serine/threonine kinase activity; Molecular Function  GO:0005524; ATP binding; Molecular Function  GO:0006468; protein phosphorylation; Biological Process |
| ssu-miR156 | willow_GLEAN_10021364 | GO:0016021; integral to membrane; Cellular Component  GO:0055085; transmembrane transport; Biological Process |
| ssu-miR396 | willow_GLEAN_10021485 | GO:0030528; transcription regulator activity; Molecular Function  GO:0045449; regulation of transcription; Biological Process |
| ssu-miR160 | willow_GLEAN_10021828 | GO:0000155; two-component sensor activity; Molecular Function  GO:0000156; two-component response regulator activity; Molecular Function  GO:0000160; two-component signal transduction system (phosphorelay); Biological Process  GO:0005524; ATP binding; Molecular Function  GO:0006355; regulation of transcription, DNA-dependent; Biological Process  GO:0007165; signal transduction; Biological Process  GO:0016020; membrane; Cellular Component  GO:0004673; protein histidine kinase activity; Molecular Function  GO:0018106; peptidyl-histidine phosphorylation; Biological Process  GO:0016772; transferase activity, transferring phosphorus-containing groups; Molecular Function |
| ssu-miR396 | willow_GLEAN_10022060 | GO:0005515; protein binding; Molecular Function  GO:0005524; ATP binding; Molecular Function  GO:0005634; nucleus; Cellular Component  GO:0016818; hydrolase activity, acting on acid anhydrides, in phosphorus-containing anhydrides; Molecular Function  GO:0045449; regulation of transcription; Biological Process |
| ssu-miR396 | willow_GLEAN_10022318 | GO:0003824; catalytic activity; Molecular Function  GO:0044237; cellular metabolic process; Biological Process  GO:0050662; coenzyme binding; Molecular Function |
| ssu-miR160 | willow_GLEAN_10022764 | GO:0003677; DNA binding; Molecular Function  GO:0005634; nucleus; Cellular Component  GO:0006355; regulation of transcription, DNA-dependent; Biological Process  GO:0009725; response to hormone stimulus; Biological Process  GO:0045449; regulation of transcription; Biological Process  GO:0046983; protein dimerization activity; Molecular Function |
| ssu-miR156 | willow_GLEAN_10023427 | GO:0003677; DNA binding; Molecular Function  GO:0005634; nucleus; Cellular Component |
| ssu-miR156 | willow_GLEAN_10023445 | GO:0003677; DNA binding; Molecular Function  GO:0005634; nucleus; Cellular Component |
| ssu-miR396 | willow_GLEAN_10023599 | GO:0005515; protein binding; Molecular Function  GO:0005524; ATP binding; Molecular Function  GO:0005634; nucleus; Cellular Component  GO:0016818; hydrolase activity, acting on acid anhydrides, in phosphorus-containing anhydrides; Molecular Function  GO:0045449; regulation of transcription; Biological Process |
| ssu-miR156 | willow_GLEAN_10024140 | GO:0005515; protein binding; Molecular Function |
| ssu-miR156 | willow_GLEAN_10024590 | GO:0006865; amino acid transport; Biological Process  GO:0015171; amino acid transmembrane transporter activity; Molecular Function  GO:0016020; membrane; Cellular Component  GO:0055085; transmembrane transport; Biological Process  GO:0006810; transport; Biological Process |
| ssu-miR160 | willow_GLEAN_10024734 | GO:0003677; DNA binding; Molecular Function  GO:0005634; nucleus; Cellular Component  GO:0006355; regulation of transcription, DNA-dependent; Biological Process  GO:0009725; response to hormone stimulus; Biological Process  GO:0045449; regulation of transcription; Biological Process  GO:0046983; protein dimerization activity; Molecular Function |
| ssu-miR7841 | willow_GLEAN_10024915 | GO:0004018; N6-(1,2-dicarboxyethyl)AMP AMP-lyase (fumarate-forming) activity; Molecular Function  GO:0005975; carbohydrate metabolic process; Biological Process  GO:0006188; IMP biosynthetic process; Biological Process  GO:0042578; phosphoric ester hydrolase activity; Molecular Function |
| ssu-miR472a | willow_GLEAN_10025076 | GO:0005515; protein binding; Molecular Function  GO:0008270; zinc ion binding; Molecular Function |
| ssu-miR156 | willow_GLEAN_10025156 | GO:0003677; DNA binding; Molecular Function  GO:0005634; nucleus; Cellular Component |
| ssu-miR396 | willow_GLEAN_10025311 | GO:0005515; protein binding; Molecular Function  GO:0005524; ATP binding; Molecular Function  GO:0005634; nucleus; Cellular Component  GO:0016818; hydrolase activity, acting on acid anhydrides, in phosphorus-containing anhydrides; Molecular Function  GO:0045449; regulation of transcription; Biological Process |
| ssu-miR172b/c | willow_GLEAN_10025485 | GO:0003723; RNA binding; Molecular Function |
| ssu-miR472a | willow_GLEAN_10025599 | GO:0000785; chromatin; Cellular Component  GO:0003676; nucleic acid binding; Molecular Function  GO:0003682; chromatin binding; Molecular Function  GO:0004386; helicase activity; Molecular Function  GO:0005515; protein binding; Molecular Function  GO:0005524; ATP binding; Molecular Function  GO:0005634; nucleus; Cellular Component  GO:0006333; chromatin assembly or disassembly; Biological Process  GO:0008270; zinc ion binding; Molecular Function GO:0003677; DNA binding; Molecular Function |
| ssu-miR162d | willow_GLEAN_10025638 | GO:0005215; transporter activity; Molecular Function  GO:0006810; transport; Biological Process  GO:0030288; outer membrane-bounded periplasmic space; Cellular Component |
| ssu-miR396 | willow_GLEAN_10025670 | GO:0005515; protein binding; Molecular Function  GO:0005524; ATP binding; Molecular Function  GO:0005634; nucleus; Cellular Component  GO:0016818; hydrolase activity, acting on acid anhydrides, in phosphorus-containing anhydrides; Molecular Function  GO:0045449; regulation of transcription; Biological Process |
| ssu-miR7841 | willow_GLEAN_10026664 | GO:0005488; binding; Molecular Function |
| ssu-miR156 | willow_GLEAN_10026780 | GO:0003677; DNA binding; Molecular Function  GO:0005634; nucleus; Cellular Component |
| ssu-miR396 | willow_GLEAN_10012253 | GO:0005515; protein binding; Molecular Function |
