# Supplementary material for: Detecting the Candidate Gender Determinants by Bioinformatic Prediction of miRNAs and Their Targets from Transcriptome Sequences of the Male and Female Flowers in Salix suchowensis
Source: Biomed Res Int. 2017 May 30;2017:9614596. doi: 10.1155/2017/9614596 (PMC5468582; doi:10.1155/2017/9614596)
Supplement: Supplementary file 1 — Figure S1: Number of targets for each miRNA in S. suchowensis. Green bars represent miRNAs common to female and male, whereas the red and blue bars correspond to miRNAs present exclusively in female or male, respectively. Figure S2: Compare the number targets in different component groups between female and male flower buds of S. suchowensis. Figure S3: Comparison of the expression levels of miRNA target genes between male and female buds of S. suchowensis. Color scales represent TPM normalized log2 transformed counts, whereas the red scales indicate high expression, and the blue scales indicate low expression. Table S1: Predicted miRNA target genes in female flower buds of S. suchowensis. Table S2: Predicted miRNA target genes in male flower buds of S. suchowensis. Table S3: GO annotation of miRNA targets identified in female flower buds of S. suchowensis. Table S4: GO annotation of miRNA targets identified in male flower buds of S. suchowensis. Table S5: Different expression of miRNA targets between female and male flower buds of S. suchowensis. Table S6: Distribution of miRNA target genes on 19 chromosomes of S. suchowensis. [file 9614596.f1.zip › Supplementary Materials/Table S5.doc]

**Table S5. Different expression of miRNA targets between female and male flower buds of *S. suchowensis*.**

| **Target name** | **miRNA-name** | **Expression (TPM)** | |
| --- | --- | --- | --- |
|  |  | **Female buds** | **Male buds** |
| willow_GLEAN_10025311 | ssu-miR396 | 1590.9 | 2377.9 |
| willow_GLEAN_10025485 | ssu-miR172b/c | 7600.6 | 7408.9 |
| willow_GLEAN_10025554 | ssu-miR396 | 851.1 | 1908.1 |
| willow_GLEAN_10025599 | ssu-miR472a | 6311.9 | 4088.1 |
| willow_GLEAN_10025638 | ssu-miR162d | 7413.9 | 3472.2 |
| willow_GLEAN_10025670 | ssu-miR396 | 4161.6 | 3110.1 |
| willow_GLEAN_10000878 | ssu-miR7841 | 7577.9 | 0.0 |
| willow_GLEAN_10002295 | ssu-miR1448 | 4368.9 | 1306.0 |
| willow_GLEAN_10011152 | ssu-miR156 | 576.4 | 0.0 |
| willow_GLEAN_10020084 | ssu-miR7841 | 4487.6 | 1668.0 |
| willow_GLEAN_10020110 | ssu-miR172b/c | 20636.5 | 17625.5 |
| willow_GLEAN_10020224 | ssu-miR172b/c | 19230.8 | 9649.7 |
| willow_GLEAN_10006738 | ssu-miR472a | 13389.1 | 7063.1 |
| willow_GLEAN_10018050 | ssu-miR172a | 14558.8 | 7057.5 |
| willow_GLEAN_10013064 | ssu-miR160 | 7329.3 | 700.5 |
| willow_GLEAN_10013066 | ssu-miR160 | 34660.0 | 28645.4 |
| willow_GLEAN_10021485 | ssu-miR396 | 36454.5 | 8012.8 |
| willow_GLEAN_10021828 | ssu-miR160 | 6682.8 | 6659.1 |
| willow_GLEAN_10026664 | ssu-miR7841 | 17031.1 | 6364.0 |
| willow_GLEAN_10026780 | ssu-miR156 | 6975.3 | 695.1 |
| willow_GLEAN_10022318 | ssu-miR396 | 8508.5 | 5450.3 |
| willow_GLEAN_10012353 | ssu-miR396 | 5615.1 | 0.0 |
| willow_GLEAN_10010327 | ssu-miR156 | 0.0 | 968.3 |
| willow_GLEAN_10008165 | ssu-miR172a | 16153.4 | 20694.8 |
| willow_GLEAN_10011581 | ssu-miR1448 | 4025.1 | 601.6 |
| willow_GLEAN_10002280 | ssu-miR472a | 7678.2 | 5355.6 |
| willow_GLEAN_10016441 | ssu-miR156 | 7692.9 | 2874.6 |
| willow_GLEAN_10006512 | ssu-miR172a | 8719.8 | 8231.5 |
| willow_GLEAN_10014755 | ssu-miR7841 | 14563.0 | 12666.3 |
| willow_GLEAN_10014833 | ssu-miR396 | 2372.3 | 2659.3 |
| willow_GLEAN_10009454 | ssu-miR396 | 2644.9 | 0.0 |
| willow_GLEAN_10023177 | ssu-miR396 | 10515.3 | 785.8 |
| willow_GLEAN_10023371 | ssu-miR6423 | 3928.5 | 0.0 |
| willow_GLEAN_10023427 | ssu-miR156 | 5191.0 | 5431.1 |
| willow_GLEAN_10023445 | ssu-miR156 | 3907.7 | 2920.3 |
| willow_GLEAN_10023599 | ssu-miR396 | 480.5 | 2154.5 |
| willow_GLEAN_10002316 | ssu-miR396 | 0.0 | 465.7 |
| willow_GLEAN_10008040 | ssu-miR396 | 7502.4 | 4077.6 |
| willow_GLEAN_10018904 | ssu-miR396 | 3983.6 | 1082.6 |
| willow_GLEAN_10018925 | ssu-miR472a | 1818.8 | 0.0 |
| willow_GLEAN_10001511 | ssu-miR472a | 14939.1 | 8283.3 |
| willow_GLEAN_10006436 | ssu-miR156 | 3052.8 | 1629.6 |
| willow_GLEAN_10010690 | ssu-miR172a | 3897.3 | 13834.9 |
| willow_GLEAN_10010741 | ssu-miR162 | 8341.6 | 20056.9 |
| willow_GLEAN_10009255 | ssu-miR169 | 10496.7 | 9208.8 |
| willow_GLEAN_10002294 | ssu-miR390 | 0.0 | 5628.2 |
| willow_GLEAN_10005685 | ssu-miR472a | 0.0 | 766.2 |
| willow_GLEAN_10019839 | ssu-miR6423 | 4589.4 | 20578.8 |
| willow_GLEAN_10019848 | ssu-miR396 | 2991.3 | 4843.5 |
| willow_GLEAN_10019875 | ssu-miR166 | 7372.7 | 3673.2 |
| willow_GLEAN_10017442 | ssu-miR1448 | 3058.8 | 5189.7 |
| willow_GLEAN_10004254 | ssu-miR156 | 27633.7 | 37917.6 |
| willow_GLEAN_10024590 | ssu-miR156 | 6263.6 | 1248.3 |
| willow_GLEAN_10024734 | ssu-miR160 | 3105.7 | 1038.9 |
| willow_GLEAN_10024780 | ssu-miR172a | 16681.3 | 1653.4 |
| willow_GLEAN_10024912 | ssu-miR172b/c | 9961.1 | 701.5 |
| willow_GLEAN_10024915 | ssu-miR7841 | 7413.9 | 1771.5 |
| willow_GLEAN_10025076 | ssu-miR472a | 5384.5 | 9657.6 |
| willow_GLEAN_10025156 | ssu-miR156 | 17743.5 | 1433.6 |
| willow_GLEAN_10003310 | ssu-miR396 | 471.3 | 352.2 |
| willow_GLEAN_10020883 | ssu-miR160 | 16681.3 | 1653.4 |
| willow_GLEAN_10020998 | ssu-miR396 | 16681.3 | 5790.7 |
| willow_GLEAN_10011083 | ssu-miR167 | 26558.3 | 25037.8 |
| willow_GLEAN_10025920 | ssu-miR396 | 5396.3 | 9073.9 |
| willow_GLEAN_10026446 | ssu-miR166 | 2513.1 | 3756.3 |
| willow_GLEAN_10005202 | ssu-miR396 | 8736.2 | 1374.5 |
| willow_GLEAN_10010173 | ssu-miR160 | 9961.1 | 701.5 |
| willow_GLEAN_10002033 | ssu-miR472a | 8202.0 | 5536.5 |
| willow_GLEAN_10022003 | ssu-miR396 | 4754.4 | 6598.7 |
| willow_GLEAN_10022060 | ssu-miR396 | 5811.4 | 2171.5 |
| willow_GLEAN_10004638 | ssu-miR166 | 6812.8 | 12626.7 |
| willow_GLEAN_10021194 | ssu-miR162 | 0.0 | 908.4 |
| willow_GLEAN_10021209 | ssu-miR172a | 5366.8 | 6684.7 |
| willow_GLEAN_10021364 | ssu-miR156 | 5456.3 | 4077.6 |
| willow_GLEAN_10009152 | ssu-miR162 | 50080.1 | 53540.7 |
| willow_GLEAN_10011703 | ssu-miR156 | 2144.4 | 3205.1 |
| willow_GLEAN_10007470 | ssu-miR472c | 3803.0 | 636.1 |
| willow_GLEAN_10001441 | ssu-miR472c | 14196.8 | 11872.9 |
| willow_GLEAN_10010398 | ssu-miR167 | 19215.5 | 14892.3 |
| willow_GLEAN_10016069 | ssu-miR396 | 4734.8 | 3033.0 |
| willow_GLEAN_10002818 | ssu-miR156 | 4856.7 | 5646.0 |
| willow_GLEAN_10016624 | ssu-miR156 | 12668.7 | 3606.8 |
| willow_GLEAN_10016755 | ssu-miR472a | 0.0 | 976.0 |
| willow_GLEAN_10006541 | ssu-miR396 | 5280.3 | 1973.1 |
| willow_GLEAN_10002936 | ssu-miR396 | 5765.9 | 12209.0 |
| willow_GLEAN_10022764 | ssu-miR160 | 7413.9 | 1771.5 |
| willow_GLEAN_10022899 | ssu-miR396 | 324.4 | 1454.4 |
| willow_GLEAN_10002393 | ssu-miR166 | 3965.1 | 4103.0 |
| willow_GLEAN_10010577 | ssu-miR472a | 5671.6 | 7417.5 |
| willow_GLEAN_10009038 | ssu-miR396 | 54796.8 | 43314.0 |
| willow_GLEAN_10003262 | ssu-miR396 | 7744.0 | 2042.6 |
| willow_GLEAN_10002635 | ssu-miR472a | 10753.2 | 2678.7 |
| willow_GLEAN_10007986 | ssu-miR172b/c | 40844.2 | 38746.7 |
| willow_GLEAN_10007998 | ssu-miR156 | 7656.9 | 2288.9 |
| willow_GLEAN_10008019 | ssu-miR156 | 14006.1 | 11101.6 |
| willow_GLEAN_10011433 | ssu-miR166 | 1342.8 | 7167.7 |
| willow_GLEAN_10019227 | ssu-miR472c | 1762.2 | 1755.9 |
| willow_GLEAN_10001273 | ssu-miR472a | 13898.0 | 8309.2 |
| willow_GLEAN_10008484 | ssu-miR1448 | 1255.9 | 0.0 |
| willow_GLEAN_10014923 | ssu-miR167 | 732.5 | 245.0 |
| willow_GLEAN_10015032 | ssu-miR172a | 11539.4 | 19163.9 |
| willow_GLEAN_10003110 | ssu-miR1448 | 2934.6 | 1462.1 |
| willow_GLEAN_10014582 | ssu-miR396 | 884.8 | 3967.4 |
| willow_GLEAN_10019627 | ssu-miR160 | 2632.0 | 1124.0 |
| willow_GLEAN_10019637 | ssu-miR172a | 3662.4 | 5474.0 |
| willow_GLEAN_10014093 | ssu-miR396 | 6295.7 | 1344.3 |
| willow_GLEAN_10013536 | ssu-miR156 | 4437.3 | 736.9 |
| willow_GLEAN_10013635 | ssu-miR396 | 4378.0 | 3271.8 |
| willow_GLEAN_10010895 | ssu-miR167 | 13726.7 | 6752.8 |
| willow_GLEAN_10001417 | ssu-miR162 | 1278.8 | 5734.2 |
| willow_GLEAN_10023982 | ssu-miR6423 | 4051.7 | 0.0 |
| willow_GLEAN_10024140 | ssu-miR156 | 0.0 | 1671.9 |
| willow_GLEAN_10015270 | ssu-miR167 | 3360.4 | 2484.9 |
| willow_GLEAN_10012461 | ssu-miR156 | 0.0 | 2422.4 |
| willow_GLEAN_10007066 | ssu-miR172a | 72878.7 | 76509.8 |
| willow_GLEAN_10020581 | ssu-miR172a | 10956.8 | 17865.5 |
| willow_GLEAN_10020659 | ssu-miR396 | 7981.4 | 6278.7 |
| willow_GLEAN_10020679 | ssu-miR7841 | 6070.0 | 5897.2 |
| willow_GLEAN_10020754 | ssu-miR1448 | 788.2 | 2945.3 |
| willow_GLEAN_10012047 | ssu-miR160 | 24135.8 | 1242.2 |
| willow_GLEAN_10006848 | ssu-miR172a | 7307.5 | 8737.8 |
| willow_GLEAN_10006870 | ssu-miR472a | 41748.2 | 28469.8 |
| willow_GLEAN_10005448 | ssu-miR396 | 1174.8 | 3511.9 |
